# PUMA reduces FASN ubiquitination to promote lipid accumulation and tumor progression in human clear cell renal cell carcinoma

**DOI:** 10.1038/s41419-025-07782-y

**Published:** 2025-06-19

**Authors:** Qianqian Luo, Qi Wang, Jian Shi, Qingyang LV, Zirui Dong, Wen Li, Yaru Xia, Jingchong Liu, Hongmei Yang

**Affiliations:** 1 Department of Pathogen Biology, School of Basic Medicine, Tongji Medical College, Huazhong University of Science and Technology, Wuhan, China; 2 Department of Urology, Union Hospital, Tongji Medical College, Huazhong University of Science and Technology, Wuhan, China

**Keywords:** Targeted therapies, Ubiquitylation

## Abstract

While the p53 upregulated modulator of apoptosis (PUMA) is traditionally recognized for promoting cell apoptosis and enhancing chemotherapy efficacy in various cancers, its role in clear cell renal cell carcinoma (ccRCC) remains unclear due to ccRCC’s chemotherapy resistance. In this study, we discover a novel oncogenic role for PUMA in ccRCC, diverging from its known apoptotic function, through assessments of public datasets, clinical tissue samples, and cell line experiments. Abnormally high expression of PUMA positively correlates with clinical stages and poor prognosis. Notably, PUMA’s role in ccRCC appears to be independent of apoptosis. Instead, it facilitates tumor progression and lipid accumulation through mechanisms involving the key metabolic regulator, fatty acid synthase (FASN). Specifically, the N44-102 amino acid sequence of PUMA, distinct from the previously studied BH3 domain, is crucial for its interaction with FASN. As a mechanism, PUMA stabilizes FASN by binding to ubiquitin-specific protease 15 (USP15), reducing FASN ubiquitination and degradation, thereby forming the PUMA-USP15-FASN axis. These findings challenge the established view of PUMA’s role in cancer biology. Furthermore, PUMA knockdown significantly inhibits tumor growth and enhances the sensitivity of ccRCC tumors to metabolic inhibition. These results position PUMA as a novel metabolic regulator and a potential therapeutic target in ccRCC. The combined inhibition of PUMA and FASN further supports the therapeutic potential of targeting this metabolic axis.

## Introduction

Renowned for its aggressive invasion and high metastasis rates, clear cell renal cell carcinoma (ccRCC) is responsible for over 90% of deaths resulting from kidney cancers [1, 2]. While ccRCC is characterized by multiple gene mutations, mutations in the *VHL* gene commonly lead to the accumulation of hypoxia-inducible factor (HIF). This leads to a series of aberrations in pathways, such as vascular endothelial growth factor (VEGF) and mTOR [3, 4]. Researchers and drug producers have targeted these mutated genes and abnormal molecules to develop therapies for ccRCC. The recent approval of the HIF-2α inhibitor Belzutifan represents a notable outcome of this research direction [5]. However, patients with ccRCC often acquire resistance to current first-line drugs, such as VEGF inhibitors, tyrosine kinase inhibitors, and mTOR inhibitors, which ultimately reduces survival [6]. These challenges highlight the need for earlier diagnosis and the identification of novel mechanisms and therapeutic targets for ccRCC.

Notably, abnormal lipid metabolism has emerged as a critical aspect of ccRCC. Lipid droplets within ccRCC cells are a key pathological feature and play a crucial role in stabilizing the endoplasmic reticulum and promoting tumor cell survival [7]. Key enzymes involved in lipid biosynthesis, such as fatty acid synthase (FASN), are upregulated in ccRCC and are linked with poor prognosis [8]. Furthermore, recent studies, including our own, have highlighted a significant link between lipid accumulation and the oncogenic factor HIF-2α, suggesting that targeting lipid metabolism pathways, including key enzymes like FASN, could provide new therapeutic opportunities for ccRCC [9–11].

The tumor suppressor gene *p53* is well known for its role in maintaining genome stability by regulating the cell cycle and apoptosis [12, 13]. Recently, it has also been shown to play a significant role in regulating lipid metabolism and cellular senescence [14, 15]. Studies on renal cancer incidence in mice have shown that combined mutations in *VHL* and *p53* can induce precursor lesions of ccRCC, underscoring the importance of p53 in this cancer type [16]. Furthermore, mutations in the Polybromo-1 (*PBRM1*) gene, the second most frequently mutated gene in renal cell carcinoma (RCC), may disrupt its interaction with activated p53. Disruption of this interaction may impair the tumor-suppressive function of *PBRM1* [17]. Following this direction, inhibiting *p53* gene mutations and enhancing its activation emerges as a promising avenue for RCC anticancer drug development. This is because the *p53* mutation rate in RCC is very low while the p53 pathway is mostly in an inhibitory state [18, 19]. p53 upregulated modulator of apoptosis (PUMA), a key effector in the p53 pathway, is known for promoting cell apoptosis in various cancers [20]. Activating the p53 pathway and reversing the disconnection of the p53/PUMA axis could mitigate the resistance of RCC to radiotherapy and chemotherapy [21, 22]. Moreover, p53 activation can promote glycolysis through PUMA [23]. However, the role of PUMA in ccRCC remains unclear, prompting further investigation into this effector.

PUMA is a member of the BH3-containing B-cell lymphoma 2 (Bcl-2) family. Its gene name is BCL2 binding component 3 (*BBC3*). PUMA is typically expressed at low levels in normal tissues and in most cancers [24]. When activated by the tumor suppressor p53 and certain transcriptional regulators, such as transfer factor nuclear factor-κB (NF-κB) and interferon regulatory factor 1 (IRF-1), PUMA induces apoptosis by binding to pro-apoptotic Bcl-2 protein via its BH3 domain [22]. This robust promotion of apoptosis has positioned PUMA as a crucial candidate gene for cancer treatment [25]. Previous studies suggest that PUMA can mediate the pro-apoptotic effect of the chemotherapy drug oxaliplatin in colon cancer and non-small cell lung cancer, serving as a biomarker to predict tumor sensitivity to pemetrexed [26]. However, the role of PUMA in cancer is complex. In some cancers, high expression of PUMA is linked with malignant tumor progression [27, 28]. In RCC, PUMA-deficient tumors are even positively associated with the highest levels of apoptotic cells [29], suggesting that PUMA might have non-apoptotic roles that promote tumor survival and progression. Our study shows that knocking down PUMA in ccRCC does not affect apoptosis levels or the activities of EndoG and CAD (Figs. S1 and S2), the key molecules in the sub-lethal signaling pathway. Moreover, high expression of PUMA can act as a requisite pro-angiogenic factor for the proliferation of vascular and glial cells [24, 30]. In terms of metabolic regulation, PUMA facilitates the metabolic shift toward aerobic glycolysis by interacting with the mitochondrial pyruvate carrier in hepatocellular carcinoma [23].

Given that ccRCC is characterized by pronounced glycolytic and lipid anabolic features, we hypothesize that PUMA can facilitate metabolism reprogramming and malignant progression in ccRCC. To investigate this hypothesis, we pose two key research questions: (1) Does PUMA exhibit cancer-promoting effects in ccRCC? (2) How does PUMA contribute to the malignant progression of ccRCC?

In this study, we conducted clinical, cellular, and animal experiments to address these questions. Using online databases and clinical tissue samples, we evaluated PUMA expression in ccRCC and its prognostic potential. Contrary to the traditional understanding of PUMA as solely a pro-apoptotic protein, our findings reveal a novel role for PUMA in driving disease progression and fatty acid synthesis in ccRCC. We discovered that high expression of PUMA stabilizes FASN through USP15, forming a PUMA–USP15–FASN axis that promotes tumor progression and lipid accumulation. This unexpected oncogenic function of PUMA identifies it as a promising therapeutic target and suggests a synergistic effect when combined with FASN inhibitors to inhibit tumor growth in ccRCC.

## Materials and methods

### Clinical samples and experimental cellular cultures

Clinical samples of ccRCC, including tumor and adjacent normal kidney tissues, were sourced from the Department of Urology at Union Hospital, affiliated with Tongji Medical College in Wuhan, China. Fresh paired clinical tissues were rapidly frozen in liquid nitrogen before they were preserved at −80 °C for subsequent immunohistochemistry analysis, as well as mRNA and protein extraction. All sample tissues were classified as ccRCC according to clinical pathological examination. To ensure ethical compliance, all clinical tissue samples were used with the full and informed consent of the patient before surgery. Ethical approval for this research was granted by the Tongji Medical College Ethics Board, Huazhong University of Science and Technology (HUST) (Supplemental Material).

Human kidney cells HK-2 (RRID: CVCL_0302) were obtained from the American Type Culture Collection (ATCC, CRL-2190) and used as the normal control group. Human renal cell carcinoma cell lines, namely A-498 (RRID: CVCL_1056) (ATCC, HTB-44), Caki-1 (RRID: CVCL_0234) (ATCC, HTB-46), 786-O (RRID: CVCL_1051) (ATCC, CRL-1932), ACHN (RRID: CVCL_1067) (ATCC, CRL-1611), and OS-RC-2 (RRID: CVCL_1626) were provided by the National Collection of Authenticated Cell Cultures (NCACC, TCHu 40), which were designated as the experimental groups. HEK293T cells (RRID: CVCL_0063) (ATCC, CRL-3216) were employed as the host cells for transfecting the plasmid. Cells were cultured in high-glucose DMEM medium (Gibco, Thermo Fisher Scientific Inc., Massachusetts, USA) supplemented with 10% fetal calf serum (HyClone, Logan, UT). Optimal growth conditions were established with a constant temperature of 37 °C and 5% CO_2_ for cell cultures.

### Plasmids and lentiviral plasmids

Plasmids pBI-EGFP-PUMA (#16590), Clover2-N1(#54537), and pEGFP-N1(#172281) were sourced from Addgene (Massachusetts, USA). To construct plasmid Clover2-PUMA, we designed and cloned specific genetic constructs using standard molecular biology techniques. The construction process involved double enzyme digestion with Bgl‖ and EcoRI restriction enzymes. The following primers were used: Upstream primer-5’ GGAAGATCTTCCATGGCCCGCGCAC 3’, Downstream primer-5’ CCGGAATTCCGGATTGGGCTCCATC 3’. The resulting plasmids were verified by full-length sequencing. Plasmids containing HA-PUMA, FLAG-PUMA, mCherry-FASN, mRuby2-FASN, FLAG-FASN, UB, HA-USP7, and HA-USP15 were sourced from Tsingke Biotechnology Co., Ltd. Transfection reagents, including Lipo3000, p3000, and Opti-MEM medium, were procured from Thermo Fisher. Gene-deficient cells were generated using CRISPR/Cas9 genome editing by transducing the cells with the plasmid vector YKO-RP003 (Ubigene Co., Ltd, Guangzhou, China) and selecting with puromycin. The guide RNAs used for gene targeting were as follows: EndoG (ACGCCGACTACCGCGGCAGT), CAD (CAGCCCGAGGAAGTTCGGCG), BAX (CTGCAGGATGATTGCCGCCG), and BAK (CCTTACCTCTGCAACCTAGC). Lentiviral vectors for PUMA knockdown (shPUMA) and the corresponding negative control (CN) were acquired from Genechem Co., Ltd (Shanghai, China). Detailed plasmid maps and sequence information are available with the authors upon request.

### In vivo xenograft model

We acquired 30 male BALB/c mice at the age of 4 weeks from Vitalstar Biotechnology Co., Ltd (Beijing, China). Mice were maintained under specific pathogen-free (SPF) conditions. To establish a subcutaneous tumor model, 5 × 10^6^ Caki-1 cells stably expressing shPUMA or CN lentiviral plasmids were injected subcutaneously into 5-week-old mice in the left armpit. The mice were randomly assigned to experimental groups. The treatment group received a weekly intraperitoneal injection of 20 mg/kg C75 inhibitor, while the negative control was administered an equal volume of normal saline. We measured the tumor sizes every 3 or 4 days after implantation, and the animals were euthanized after 30 or 42 days. Subcutaneous tumor tissues were excised, weighed, snap-frozen in liquid nitrogen, and stored at −80 °C. Half of the excised tissues underwent paraffin and frozen sections, subjected to staining with hematoxylin and eosin (H&E), immunohistochemistry, and Oil Red O (ORO) to facilitate inter-group comparisons. The analysis of these tissues was performed blinded to the experimental group allocation. The immunohistochemistry process involved the use of antibodies against PUMA, FASN, Ki67, Caspase-3/cleaved Caspase-3, Caspase-9, and cleaved Caspase-9. Antibody information is listed in Supplementary Table S1. For the tail vein metastasis tumor model, 2 × 10^6^ A-498 cells stably expressing shPUMA or CN lentiviral plasmids were then injected into the mice’s tail vein. After 40 days, the animals were euthanized to collect metastatic liver and lung tissues. The pathological evaluation of these metastatic tissues was also performed blinded to the group allocation. H&E staining was conducted for an assessment of the pathological conditions of the metastatic foci. This study was approved by the HUST Experimental Animal Ethics (IACUC 3617, in Supplemental Material).

### Western blotting analysis

Proteins were extracted from tissues and cells using ice-cold protein lysis buffer (RIPA, P0013C, Beyotime, Shanghai, China) supplemented with 100 μg/mL PMSF (ST507, Beyotime) and phosphatase inhibitor cocktail (B14001, Bimake, Delhi, India). Lysates were vortexed three times and then centrifuged at 12,000 rpm for 15 min at 4 °C. The supernatant was collected, and protein concentration was measured using a BCA assay. Equal amounts of protein were mixed with loading buffer, denatured at 95 °C for 10 min, and stored at –20 °C until use. Samples were separated by SDS–PAGE and transferred onto PVDF membranes (Roche, Mannheim, Germany). Membranes were blocked and incubated overnight at 4 °C with primary antibodies, followed by washing with PBST and incubation with secondary antibodies for 2 h at room temperature. Immunoreactive bands were visualized by chemiluminescence and quantified. Details of the antibodies used are listed in Supplementary Table S1.

### Real-time quantitative PCR (qPCR) analysis

Total RNA was extracted from tissue and cell samples using Trizol reagent (Thermo Fisher Scientific), following the manufacturer’s protocols. The extracted RNA was subsequently reverse-transcribed using a cDNA Synthesis SuperMix (11141ES, YEASEN) and then analyzed by quantitative PCR with SYBR Master Mix (11171ES, YEASEN). Gene expression levels of PUMA and FASN were normalized to β-actin. Relative gene expression levels were calculated using the 2^−ΔΔCT^ method. Each experiment was repeated at least three times. Primer sequences are listed in Supplementary Table S2.

### Cellular compartment protein isolation

Cellular fractionation was performed using the Nuclear and Cytoplasmic Protein Extraction Kit (P0028, Beyotime) following the manufacturer’s protocol. This procedure enabled the precise isolation of cytoplasmic and nuclear proteins, facilitating the targeted assessment of PUMA’s intracellular localization in ccRCC cells.

To investigate subcellular components in detail, we isolated mitochondria and the remaining cytoplasmic proteins using the Mitochondria Isolation Kit (#89874, Thermo Fisher Scientific). The isolated protein fractions were promptly processed for Western blot verification to confirm the effectiveness of mitochondrial isolation and the presence of PUMA.

### Immunohistochemistry and immunofluorescence

As an analysis of clinical pathological tissues and subcutaneous tumors in nude mice, we performed immunohistochemistry following previously published procedures [9]. Tissue specimens were processed with sequential fixation, dehydration, and paraffin embedding. The subsequent sections were prepared and subjected to immunohistochemistry. Afterward, the tissue sections underwent incubation with the primary antibodies, followed by subsequent incubation with the corresponding secondary antibodies. We obtained random images of the processed tissue sections for the assessment of protein expression and localization. The analysis was blinded to the sample group allocation during image analysis.

In vitro experiments involved the assessment of subcellular localization for PUMA and FASN using immunofluorescence staining. The procedure was conducted according to previously published protocols [31]. Cells were fixed, permeabilized, blocked, and then incubated with primary antibodies, followed by fluorescent secondary antibodies. Images were captured using a fluorescence microscope (Leica, Wetzlar, Germany). Antibody information is provided in Supplementary Table S1.

### Transwell assay

Cells underwent a 24-h starvation period and were uniformly seeded in transwell chambers to induce migration and invasion through a serum-containing medium. After a 24-h incubation, cells were fixed with methanol for 30 min, followed by a 20-min staining with 0.05% crystal violet. Images were acquired from randomly selected fields. Chambers lacking Matrigel coating were utilized for assessing ccRCC cell migration, while Matrigel-coated chambers were employed for invasion experiments. Invasion chambers were pre-coated with matrix gel (dilution 1:8, Corning Inc., New York, US) one hour before the assay. The cell density for migration analysis was 1 × 10^5^ cells, and for invasion analysis, it was 2 × 10^5^ cells.

### Cell viability assay and drug combination effect experiment

The CCK-8 Kit (40203ES, YEASEN) assay was performed to assess cell viability and proliferation systematically, providing an optical density (OD) value that reflects cell metabolic activity and indicates cell growth. Cells in active growth and exhibiting exponential growth were selected, seeded (2000 per well) in a 96-well plate, and incubated. Every 24 h, 10 µL CCK-8 reagent was mixed with 90 µL cell culture medium and added to each well, including blank controls. After a 2 h incubation, we measured the absorbance at 450 nm using a microplate reader (INFINITE 200 PRO, TECAN, Mannedorf, Switzerland).

For the drug combination effect experiment, we seeded cells (10,000 per well) in a 96-well plate and incubated for 24 h. Following this, various concentrations of drugs were added to each well. After another 48 h incubation, cell viability was assessed by measuring absorbance at 450 nm.

### Colony formation assay

Colony formation assays were employed to comprehensively assess cellular proliferative capacity. A total of 1000 cells per well were seeded in six-well plates. After a 14-day incubation, cells were fixed with methanol for 30 min, followed by a 20-min staining with 0.05% crystal violet. Colonies were then observed and imaged, followed by colony formation analysis using ImageJ software.

### Oil red O (ORO) staining

Cells were seeded in a 12-well plate and cultured to 30% confluence. ORO working solution was prepared by mixing six parts saturated ORO dye with four parts distilled water, followed by filtration through a 0.45 μm filter (G1015, Servicebio, Wuhan, China). Cells were fixed with 4% paraformaldehyde for 10 min and then stained with the ORO working solution at room temperature for 30 min. Images were captured using a microscope. For quantitative analysis, 250 μL of isopropanol was added to extract lipid droplets. After a 5 min incubation at room temperature, absorbance was measured at 510 nm. The same procedure was applied for fresh-frozen tissue sections to ensure consistency.

### Periodic acid-Schiff (PAS) staining

Cells stably expressing shPUMA or CN lentiviral plasmids were cultured in 24-well plates until 50% confluence. PAS staining was performed using a commercial kit (C0142S, Beyotime) according to the manufacturer’s instructions. Cells were first fixed with 70% ethanol for 10 min. Next, a 100 μL periodic acid solution was added, and cells were incubated in a humid chamber in the dark for 10 min. For negative control, periodic acid was omitted. Afterward, 100 μL of Schiff reagent was added, and cells were stained at 37 °C in the dark for 30 min. Following staining, cells were rinsed with distilled water for 5 min, counterstained with 100 μL of hematoxylin for 30 s, and rinsed again. Images were captured under a microscope to compare glycogen or polysaccharide content (appearing red or purple) between groups.

### Triglyceride and total cholesterol assay

Cells were cultured in 6 cm dishes until reaching ~90% confluence. Cell suspensions were prepared following the protocol of the Triglyceride Assay Kit (A110-1-1, Nanjing Jiancheng Bioengineering Institute, Nanjing, China). The suspensions were centrifuged at 1000 rpm for 10 min, and the supernatant was discarded. The cell pellet was washed three times with PBS and then lysed in 200 µL of 2% Triton X-100 (TB200, Solarbio, Beijing, China) for 40 min. Next, 2.5 µL of the lysate was mixed with 250 µL of the working reagent and incubated at 37 °C for 10 min. A small aliquot of the sample was reserved for protein quantification. Absorbance was measured at 546 nm using a microplate reader (INFINITE 200 PRO, TECAN, Mannedorf, Switzerland). All measurements were performed in triplicate to ensure reproducibility. The triglyceride content (mmol/gprot) was calculated using the following formula:

$${{\rm{Triglyceride}}\,{\rm{content}}}={\rm{C}}{{\_}}{\rm{std}}\times ({\rm{A}}{\_}{\rm{meas}}-{\rm{A}}{{\_}}{\rm{blank}})/({\rm{A}}{{\_}}{\rm{std}}-{\rm{A}}{{\_}}{\rm{blank}})/{\rm{Cpr}},$$

where C_std is the concentration of the standard solution (mmol/L), A_meas is the absorbance of the sample, A_blank is the absorbance of the blank control, A_std is the absorbance of the standard solution, and Cpr is the protein concentration of the sample (gprot/L).

Total cholesterol was quantified following the protocol of the Total Cholesterol Assay Kit (A111-1-1, Nanjing Jiancheng Bioengineering Institute, Nanjing, China), according to the same procedure described above, except that absorbance was measured at 500 nm.

### Immunoprecipitation (IP) and mass spectrometry (MS) analysis

A total of 1 × 10^7^ cells were lysed in RIPA buffer (P0013B, Beyotime) supplemented with 100 μg/mL PMSF and 100 μg/mL protease inhibitor cocktail to obtain total protein lysates. The lysates were divided into two portions and subjected to immunoprecipitation using either IgG or PUMA antibody, followed by a magnetic bead-assisted protein complex isolation. These complexes were sent to GeneCreate Biological Engineering Co., Ltd. (Wuhan, China) for gel electrophoresis, protein bands collection, as well as liquid chromatography–tandem mass spectrometry (LC–MS/MS) analysis on enzymatically digested bands. We then obtained the proteins that interacted with PUMA and IgG. Bioinformatics data analysis criteria were set at a confidence level of ≥ 95%, including only proteins with at least one unique peptide segment. Antibody details are provided in Supplementary Table S1.

### Co-immunoprecipitation (Co-IP) assay

Stable and actively growing 1 × 10^7^ cells were collected. They were lysed in cell lysis buffer (P0013, Beyotime) for the extraction of cellular proteins. The lysate obtained was pre-cleared on magnetic beads for 10 min. Following this, the lysate was incubated with specific antibodies overnight at 4 °C, including IgG, PUMA, FASN, HA, FLAG, and USP15. After the initial incubation, magnetic beads were added to the protein–antibody complexes and further incubated at 4 °C for 4 h. The obtained immunocomplexes were then subjected to Western blotting using relevant antibodies for detection. For exogenous Co-IP assays, HEK293T cells were transfected with plasmids encoding EGFP-PUMA, HA-PUMA, FLAG-FASN, HA-PUMA ΔBH3, HA-PUMA ΔMLS, HA-PUMA ΔBH3/MLS, HA-PUMA-N43, HA-PUMA-N44–102, HA-PUMA-N103–140, UB, HA-Vector, HA-USP7, or HA-USP15. After a 48-h incubation post-transfection, cells were subjected to unified Co-IP analysis and identification following the same protocol described above.

### Fluorescence resonance energy transfer (FRET) analysis

The FRET analysis was performed to assess the direct interaction between PUMA and FASN in live cells. Plasmids were prepared: PUMA was labeled with the donor fluorophore Clover2 and FASN was labeled with the acceptor fluorophore mRuby2. HEK293T cells were seeded onto glass coverslips and cultured for 24 h. The cells were then co-transfected with the Clover2-PUMA and mRuby2-FASN plasmids and incubated for 48 h. Subsequently, glass coverslips containing the transfected cells were mounted onto an inverted single-photon laser scanning confocal microscope (LSM780, Zeiss, Germany). A ×63 oil-immersion objective was used to capture high-resolution fluorescence images. Excitation wavelengths of 488 and 559 nm were used for Clover2 and mRuby2, respectively, with emission signals collected at 500–530 and 590–670 nm to capture donor and acceptor fluorescence intensities. Image analysis and FRET efficiency quantification were then performed using MATLAB (RRID:SCR_001622)., A negative control group consisting of HEK293T cells co-transfected with Clover2 and mRuby2-FASN plasmids was included to ensure specificity.

Fluorescence intensity maps were generated for the donor (Clover2), acceptor (mRuby2), and FRET signals. To account for known crosstalk coefficients between donor and acceptor, background-subtracted fluorescence intensities for donor channel fluorescence intensity (IDD), acceptor channel fluorescence intensity (IAA), and FRET fluorescence intensity (IDA) were calculated. Normalized FRET (N-FRET) was calculated using the equation: NFRET = (IDA−aIAA−dIDD)/IDD × IAA, where *a* = 0.0174 and *d* = 0.1729.

### Protein stability assay

This approach aims to uncover how variations in PUMA levels influence the stability of FASN. We used a control group and the PUMA knockdown group. Both groups were treated with cycloheximide (CHX, 25 μM), and cells were harvested at 0, 8, 16, and 24 h post-treatment. FASN protein levels were assessed by Western blotting at each time point.

### Statistical analyses

Data analysis was performed using SPSS 22.0 (RRID:SCR_002865) and GraphPad Prism 8.02 (RRID:SCR_002798). One-way ANOVA and two-tailed Student’s *t*-tests were used for normally distributed datasets. Log-rank (Mantel–Cox) tests were performed to compare survival curves. Results are presented as means ± SEM from three independent experiments, with statistical significance indicated by *p*-values: *p < 0.05, **p < 0.01, and ***p < 0.001.

## Results

### High expression of PUMA in ccRCC correlates positively with clinical stages

**Fig. 1:**
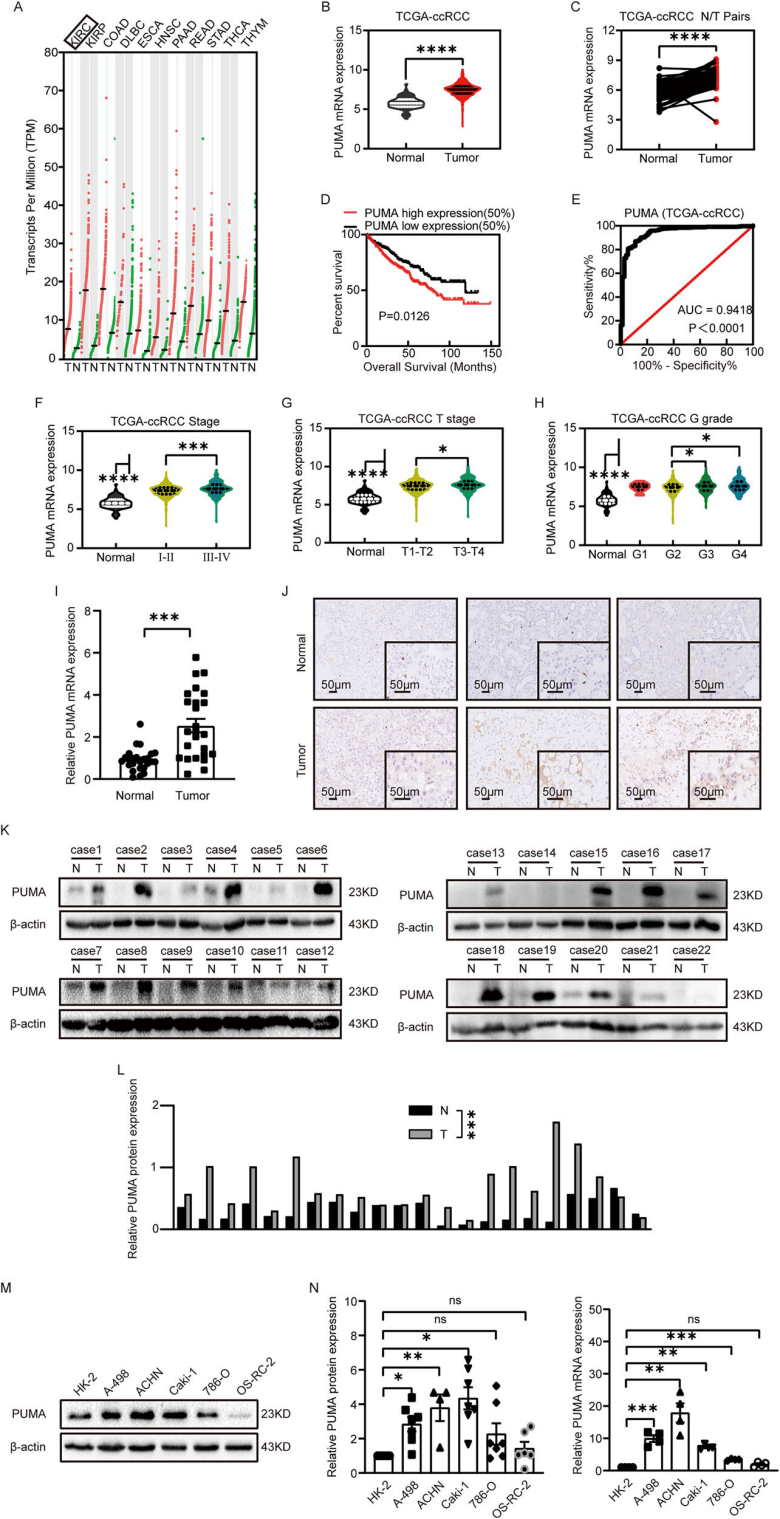
High expression of PUMA in ccRCC correlates positively with clinical stages. **A** A dot map illustrates PUMA gene expression in 11 cancer types. Red dots denote tumor tissues (T), while green dots are for the paired normal tissues (N). The fold increase in median expression for each cancer type and the sample sizes are stated as follows: KIRC (2.94, T:523, N:100), KIRP (5.49, T:286, N:60), COAD (7.75, T:275, N:349), DLBC (2.29, T:47, N:337), ESCA (3.89, T:182, N:286), HNSC (22.54, T:519, N:44), PAAD (2.71, T:179, N:171), READ (2.18, T:92, N:318), STAD (2.53, T:408, N:211), THCA (2.71, T:512, N:337), and THYM (2.29, T:118, N:339). Full names of cancer-type abbreviations are listed in Supplementary Table S3. **B** The comparison of mRNA expression of PUMA between ccRCC tissues (*n* = 534) and normal tissues (*n* = 72). **C** The mRNA expression of PUMA in 72 paired ccRCC tissue samples. Data in **B** and **C** is from TCGA-KIRC databases. **D** The Kaplan–Meier survival analysis on the ccRCC patients (*n* = 534) between high-expression and low-expression groups of PUMA. High expression was defined as PUMA mRNA levels above the median value of 7.5085 in 534 ccRCC samples, with levels below the median categorized as low expression. **E** The ROC analysis of PUMA mRNA expression levels was compared between ccRCC tumor tissues and normal kidney tissues using TCGA dataset analysis (AUC = 0.9418). AUC: areas under the curve. **F** and **G** The PUMA mRNA expression, compared to matched normal samples, varies across different pathological stages, as represented by a violin plot. The mean values for each group are: normal (5.787, *n* = 72), stages I–II (7.346, *n* = 309), stages III–IV (7.637, *n* = 206), T1–T2 (7.422, *n* = 342), and T3–T4 (7.602, *n* = 191). In **H**, PUMA mRNA expression in patient normal tissues is compared to different grading (G grade) using a violin plot. The average expression in tumors ranges from 7.449 to 7.672, showing a fold increase of 1.29 to 1.33 compared to normal tissues (G in tumors: *n* = 14, 228, 207, 81). **I** The mRNA expression of PUMA in a set of tissue samples, comprising both adjacent normal tissues and tumor tissues, *n* = 25. **J** Immunohistochemical staining of PUMA in paired tumor and adjacent non-cancerous tissues, *n* = 6. The scale bar is 50 μm. **K** Western blot presents PUMA protein expression in ccRCC tumor tissues (T) and their corresponding adjacent normal tissues (N). Subsequent quantitative analysis of PUMA protein expression is depicted in the histogram (**L**). The sample size is 22. **M** Western blot depicting the protein expression of PUMA in various human ccRCC lines (A-498, ACHN, Caki-1, 786-O, OS-RC-2) with the HK-2 as the control group. Statistical analysis on PUMA protein and mRNA expression in the histogram (**N**). The mean ± SEM from three or more repeated experiments is presented (*p < 0.05, **p < 0.01, ***p < 0.001, ns = not significant). The denoting system is applied to the tables and figures throughout this study.

We accessed the mRNA data of PUMA from GEPIA and UCSC Xena data hub [32, 33]. A gene dot map analysis from GEPIA revealed high levels of PUMA expression in 11 cancer types compared to normal tissues (see Fig. 1A), including ccRCC (referred to as Kidney Renal Clear Cell Carcinoma, KIRC). A violin plot and a line diagram also showed high levels of PUMA mRNA in ccRCC tumors (Fig. 1B, C), based on data from 534 ccRCC patients and 72 paired tissue samples (tumor and corresponding normal kidney tissue). High PUMA expression was defined as RNA levels above the median value (7.5085) from 534 ccRCC samples in the TCGA dataset, with samples below the median categorized as low-expression (Fig. 1D). Some Kaplan–Meier curve analyses suggested a potential association between high PUMA expression and shorter overall survival (Figs. 1D and S1A). The area under the curve (AUC) value was compared between ccRCC tumor tissues and normal kidney tissues, indicating that the expression levels of PUMA are associated with high specificity and clinical diagnostic value (Fig. 1E). The violin plot confirmed that high expression of PUMA was associated with advanced tumor staging and cancer grading in ccRCC (Fig. 1F–H). Univariate Cox analysis shows that PUMA mRNA levels are significantly associated with multiple clinicopathological parameters, including the TNM stage (Table 1). However, multivariate Cox analysis indicates that PUMA is not an independent prognostic marker (*p* = 0.194, Table 2).

**Table 1:** The mRNA expression of PUMA and clinicopathological parameters of ccRCC patients.

Parameter	Number	PUMA mRNA expression		*P*
		Low (*n* = 266)	High (*n* = 267)	
*Age (years)*				
<60	246	122	124	0.931
≥60	287	144	143	
*Gender*				
Female	188	102	86	0.148
Male	345	164	181	
*T stage*				
T1 or T2	342	182	160	0.047
T3 or T4	191	84	107	
*N stage*				
N0 or Nx	517	257	260	0.623
N1	16	9	7	
*M stage*				
M0 or Mx	452	234	218	0.051
M1	79	31	48	
*G grade*				
G1 or G2 or Gx	242	134	108	0.029
G3 or G4	288	131	157	
*TNM stage*				
I + II	324	176	148	0.01
III + IV	206	88	118	

Correlation statistics are based on clinicopathological characteristics and PUMA expression levels. Correlation statistics are generated using Pearson’s *χ*^2^ test.

**Table 2:** Univariate and multivariate Cox regression analyses of PUMA mRNA levels and patient overall survival.

Variable	Univariate analysis			Multivariate analysis		
	HR	95% CI	*P*	HR	95% CI	*P*
*Overall survival* (*n* = 533)						
*PUMA*						
Low (*n* = 266)	1.481	1.094–2.004	0.011	1.226	0.902–1.667	0.194
High (*n* = 267)						
*Age*						
<60 (*n* = 246)	1.82	1.33–2.491	0.000	1.557	1.131–2.145	0.007
≥60 (*n* = 287)						
*Gender*						
Female (*n* = 188)	0.945	0.695–1.286	0.719	0.896	0.653–1.230	0.497
Male (*n* = 345)						
*T stage*						
T1 or T2 (*n* = 342)	3.164	2.339–4.281	0.000	1.654	1.154–2.370	0.006
T3 or T4 (*n* = 191)						
*N stage*						
N0 or NX (*n* = 517)	3.862	2.091–7.132	0.000	2.475	1.322–4.634	0.005
N1 (*n* = 16)						
*M stage*						
M0 or MX (*n* = 452)	4.387	3.222–5.973	0.000	2.604	1.820–3.726	0.000
M1 (*n* = 79)						
*G grade*						
Gx or G1 or G2 (*n* = 242)	2.576	1.835–3.617	0.000	1.672	1.163–2.404	0.006
G3 or G4 (*n* = 288)						

The table presents univariate and multivariate analyses using Cox proportional hazard regression model. Hazard ratios (HR), confidence intervals (CI), *p*-values are stated. Multivariate models include parameters of T, N, M stage, G grade, age, and gender.

To further validate our findings, we examined 25 paired clinical tissue samples from ccRCC patients. The results of real-time quantitative PCR (qPCR) analysis pointed to a significant increase in PUMA mRNA expression in tumor tissues compared to that in normal tissues (Fig. 1I). Immunohistochemical analysis confirmed, again, the high expression of PUMA in tumor tissues (Fig. 1J). Furthermore, Western blot analysis showed high PUMA protein expression in tumors compared to normal tissues in 22 paired tissue samples (Fig. 1K, L). In addition, high expression of PUMA was found in human ccRCC cells (A-498, ACHN, Caki-1) as compared to human kidney cells (HK-2), both in protein and mRNA expression (Fig. 1M, N). Notably, PUMA mRNA expression was significantly higher in 786-O cells compared to HK-2, while there were no significant differences in protein levels between 786-O and OS-RC-2 (*p* > 0.05). A-498 served as a typical ccRCC cell line, and Caki-1 represented metastatic ccRCC. These two cell lines exhibited distinct mutation patterns in *VHL* and *p53* [34]. We chose them for comparison in subsequent experiments to emphasize the generality of the results. In summary, all the assessments based on public data, clinical tissues, and cell lines confirmed that PUMA is in a high expression in ccRCC, which was also associated with patients’ higher clinical stages.

### PUMA’s novel oncogenic role in ccRCC independent of apoptosis

To investigate the biological role of highly expressed PUMA in ccRCC, we transfected A-498 and Caki-1 cells with a lentiviral plasmid carrying PUMA-specific short hairpin RNA (shPUMA) to establish stable PUMA knockdown cell lines. The knockdown efficiency exceeded 70%, as confirmed by Western blotting and qPCR. The control group (CN) was generated using a blank lentiviral plasmid (Fig. S1B, C). Key apoptotic pathway molecules were measured to assess the correlation between PUMA expression and apoptosis, including full-length PARP, Bcl-2, and caspases-3, -6, and -7, as well as mitochondrial apoptosis markers such as cytochrome-C. The Western blot results (Fig. S1D, E) showed no significant differences in apoptotic marker expression or activation between the control and shPUMA groups, in both whole-cell lysates and cytosolic/mitochondrial fractions. Moreover, cytochrome-C is retained in mitochondria, further supporting the absence of mitochondrial apoptosis activation. A comparison of multiple apoptosis marker proteins confirmed that PUMA levels do not directly correlate with apoptotic activity in our model.

**Fig. 2:**
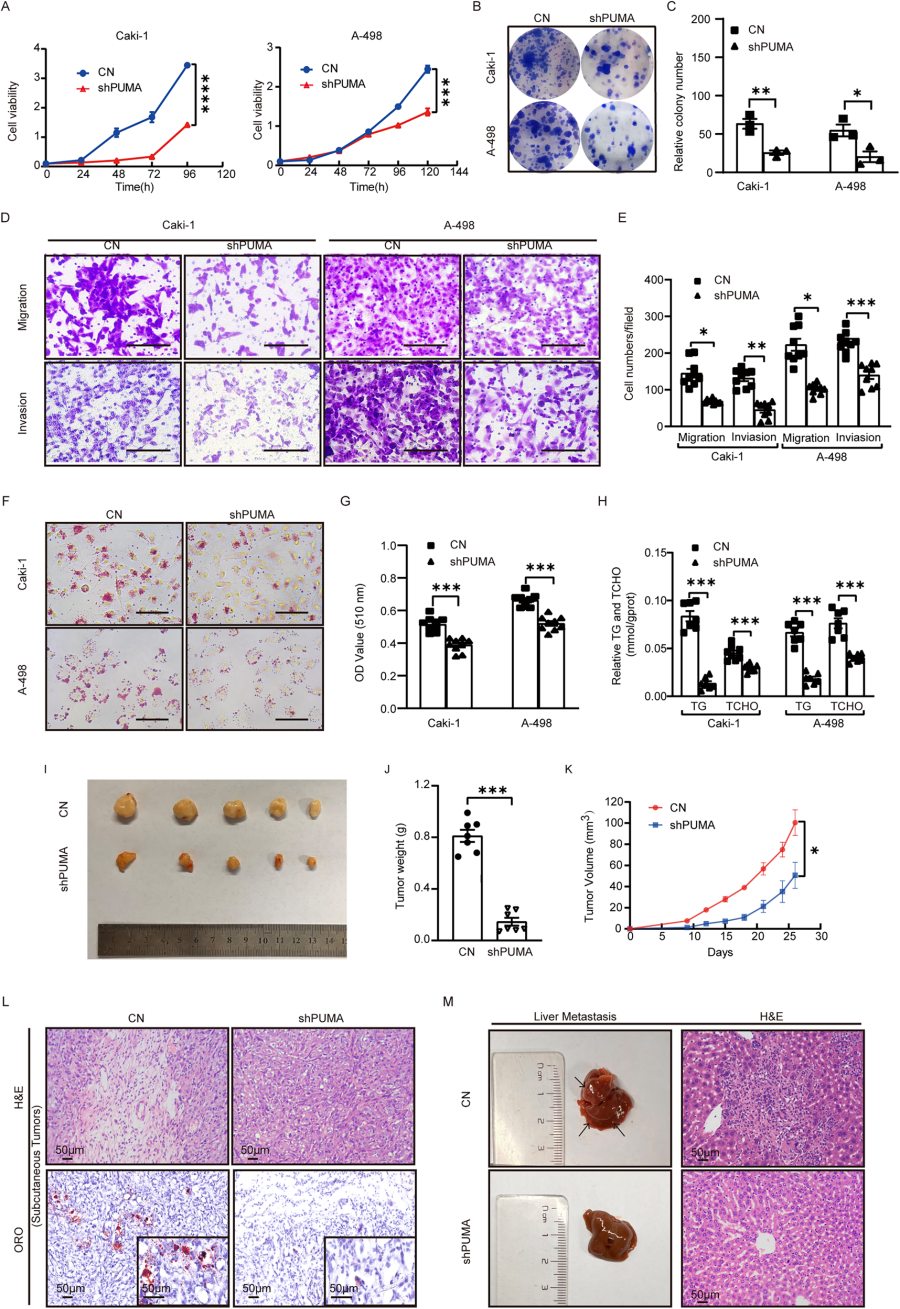
PUMA’s novel oncogenic role in ccRCC independent of apoptosis. **A** Comparison of cell proliferation curves between the shPUMA group and control group (CN) group in A-498 and Caki-1 cells. Cell viability, measured by optical density (OD) values, provides an estimate of the number of living cells, as metabolic activity is proportional to cell number. **B** Graphical representation of colony formation experiments for both the shPUMA and CN groups, accompanied by statistical analysis (**C**) (*n* = 3). **D** Microscopic images show reduced cell migration and invasion in A-498 and Caki-1 cell lines, as shown in the shPUMA group compared to the CN group. Scale: 20 μm. Statistical analysis is presented in accompanying bar graphs (**E**) (*n* = 9). **F** ORO staining results show lipid droplets in the CN group and the shPUMA group. Scale bar is 20 μm. **G** Bar graphs present statistical analysis, comparing cellular lipid droplet content between the shPUMA and CN groups. The comparison of triglycerides content and total cholesterol content is shown in (**H**) (*n* = 9). **I** Tumor size was measured at regular three-day intervals (**K**), extending until the 26th day. Subcutaneous tumor tissues were collected for image documentation and weight measurement (**J**) (*n* = 7). A *t*-test was performed, and error bars indicated the mean ± SEM. **L** H&E and ORO staining of subcutaneous tumor tissues in both the shPUMA and CN groups, with the images having a scale of 50 μm. **M** The shPUMA and CN groups in A-498 cells were intravenously injected into nude mice, as an establishment of a metastasis model. After 40 days, differences in invasive capabilities were evaluated by examining the number of liver tissue metastatic foci in both groups of mice and through H&E staining (scale: 50 μm).

With the aim of investigating whether PUMA affected ccRCC progression, we conducted colony formation experiments and cell viability tests on the PUMA knockdown group and the control group. Results indicated that compared to the control group, the cell proliferation rates and colony formation ability in the PUMA knockdown group significantly decreased in A-498 and Caki-1 cell lines (Fig. 2A–C). Transwell assays reveal that cell migration and invasion abilities were weakened in the PUMA knockdown group compared to the control group (Fig. 2D, E). In conclusion, the knockdown of PUMA was linked to a notable decrease in the malignant progression of A-498 and Caki-1 cell lines.

The malignant progression of ccRCC is fueled by metabolic reprogramming, characterized by substantial accumulation of glycogen and lipid droplets in the cytoplasm [35]. As depicted in Fig. S1F, PUMA is not directly associated with glucose accumulation in ccRCC. However, we observed a positive correlation with lipid droplets. The PUMA knockdown group showed a reduced lipid droplet content in both A-498 and Caki-1 cell lines, in contrast to the control group. Results were presented by the ORO staining and quantitative analysis (Fig. 2F, G). Compared to the control group, triglycerides and total cholesterol in both A-498 and Caki-1 cells were decreased in the PUMA knockdown group (Fig. 2H), indicating a distinctive pattern in the context of ccRCC. Conversely, in the PUMA overexpression group, cell proliferation, lipid droplets, triglycerides, and total cholesterol increased (Fig. S2A–D). The findings implied that PUMA functions as a promoter of both ccRCC progression and lipid accumulation.

To confirm our previous in vitro findings, we created subcutaneous and metastatic tumor models in nude mice. Subcutaneous and tail vein injections were performed in both PUMA knockdown and control groups. Gross tumor images and growth curves showed that tumor size, weight, and growth rate were reduced in the PUMA knockdown group, in contrast to the control group (Fig. 2I–K). Immunohistochemistry showed significantly lower PUMA expression in the knockdown group, with no difference in apoptosis markers between groups (Fig. S2E). H&E and ORO staining consistently revealed that tissue sections in the PUMA knockdown group were less malignant and exhibited lower lipid accumulation than those from the control group (Fig. 2L). In the gross liver map, the PUMA knockdown group exhibited fewer liver metastatic foci, in contrast to the control group (Fig. 2M). In general, we further consolidated our previous findings (Fig. 2A–H) that PUMA promotes the accumulation of tumor progression and lipids in ccRCC, independently of its role in apoptosis.

Recent studies highlight the activation of the mitochondrial apoptotic pathway as a driver of tumor growth and metastasis [36, 37]. This raises the possibility that PUMA could contribute to aggressive tumor behavior in ccRCC through sub-lethal signaling. Following the previous methodology [36], we assessed EndoG levels in mitochondrial and nuclear fractions. No significant nuclear translocation or activation of EndoG was observed in the shPUMA group compared to the control group (Fig. S2F). Similarly, analysis of CAD activity, via ICAD (the inhibitor of CAD) and γH2AX (a DNA damage marker), showed no significant differences between the shPUMA and control groups (Fig. S2G). These results suggest that sub-lethal signaling via EndoG or CAD is not involved in the observed tumor phenotype. We then generated ENDOG/CAD and BAK/BAX double-knockout (DKO) cells using CRISPR-Cas9 to validate the knockout efficiency via Western blotting (Fig. S2H, J). Assessment of tumor cell growth in these DKO cells showed no phenotypic differences between the shPUMA and control groups (Fig. S2I, K). Thus, the aggressive growth phenotype associated with PUMA is independent of sub-lethal signaling through EndoG/CAD or its pro-apoptotic function via BAX/BAK, suggesting that PUMA exerts its effects through alternative mechanisms.

### Direct interaction: FASN as key to PUMA’s function

To explore the subcellular localization of PUMA in ccRCC, we analyzed its expression in distinct cellular compartments using cytoplasmic-nuclear fractionation and mitochondrial isolation assays. Western blotting revealed that PUMA was primarily localized in the mitochondria of HK-2, A-498, Caki-1, and 786-O cell lines (Fig. S3A, B). Immunofluorescence imaging confirmed cytoplasmic PUMA localization and its colocalization with mitochondrial markers (Fig. S3C, D), consistent with previous studies [20]. These findings suggest that PUMA may be involved in lipid metabolism in ccRCC, given the pivotal role of mitochondria in tumor metabolic reprogramming, particularly in lipid metabolism [38].

To further explore the molecular interactions of PUMA within ccRCC, we performed IP experiments using PUMA antibodies to isolate proteins from both whole-cell and cytoplasmic lysates. Analysis of whole-cell protein showed that 320 proteins were pulled down with the PUMA antibody, compared to 41 proteins with the IgG antibody (Fig. S4A), suggesting that PUMA interacts with a distinct protein pool. Based on the IP and MS results, we performed gene ontology (GO) functional enrichment and the Kyoto Encyclopedia of Genes and Genomes (KEGG) pathway analyses. Results indicated that PUMA-related molecules were enriched in the metabolic pathway and lipid metabolism pathway (Fig. S4B, C). To improve detection sensitivity, cytoplasmic fractionation was performed to isolate proteins from the cytoplasm. Immunoprecipitation with PUMA antibodies yielded 222 proteins from the cytoplasmic fraction, compared to 139 proteins pulled down with IgG (Fig. S4D). By intersecting datasets from whole-cell IP, cytoplasmic IP, and their respective IgG controls, 43 potential targets were identified (Fig. S4E). The further intersection of the cytoplasmic IP dataset with lipid metabolism data, we discovered 28 molecules linked to lipid metabolism (Fig. S4F). Notably, these 28 molecules are part of the 43 targets from the whole-cell and cytoplasmic datasets, validating their relevance in lipid metabolic pathways. In our analysis, FASN demonstrated the highest combined score among molecules associated with lipid metabolism pathways (Fig. S4G). Given that mitochondria supply acetyl-CoA and citrate for fatty acid synthesis [39], and FASN utilizes acetyl-CoA as its substrate for fatty acid biosynthesis, it is reasonable to propose that FASN is the key downstream target of PUMA involved in lipid metabolism.

**Fig. 3:**
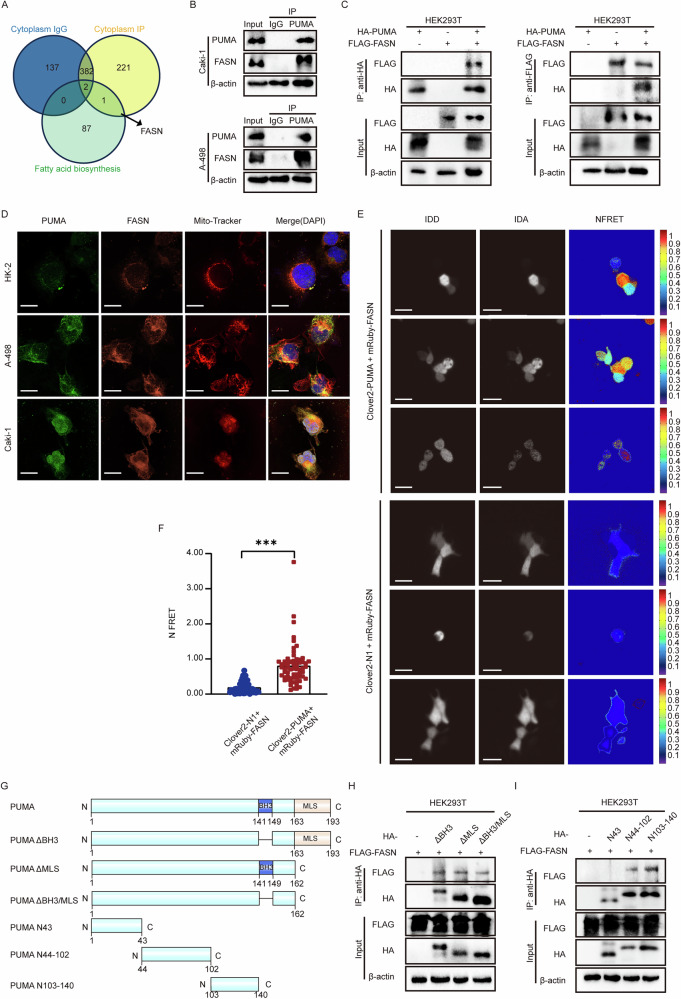
Direct interaction: FASN as key to PUMA’s function. **A** Intersection analysis using Venn diagrams was conducted on the cytoplasmic protein and fatty acid biosynthesis data sets immunopurified with PUMA antibodies. The fatty acid biosynthesis data set is sourced from the laboratory’s self-built storage library. **B** IP results demonstrate the interaction between endogenous PUMA and FASN in ccRCC cell lines (A-498, Caki-1) (*n* = 6). **C** Co-transfection of HA-tagged PUMA and FLAG-tagged FASN plasmids into HEK293T cell. IP using HA and FLAG antibodies facilitated the purification of HA-PUMA and FLAG-FASN, respectively. The interaction between PUMA and FASN was subsequently confirmed using both the FLAG antibody and HA antibody in western blot analysis (*n* = 6). **D** Confocal images of PUMA and FASN staining in HK-2, A-498, and Caki-1 cells, utilizing PUMA antibody (green), FASN antibody (orange), mitochondrial dye (Mito-Tracker Red) and DAPI (blue). Merged images illustrate the overlay of the four fluorescent channels. Scale: 5 μm. **E** HEK293T cells were co-transfected with Clover2-PUMA, and mRuby-FASN. After full crosstalk correction, fluorescence intensities for IDD (donor emission with donor excitation), IDA (acceptor emission with donor excitation), and normalized FRET (NFRET) were calculated and shown. Scale: 5 μm. Statistical histogram derived from NFRET data (*n* = 12, ***p < 0.001) demonstrated the direct interaction between PUMA and FASN, with the co-transfection of Clover2-N1 and mRuby-FASN serving as the control group (**F**). **G** Structure diagrams of plasmid constructs for various PUMA variants, including full-length, domain-deleted (PUMA ΔBH3, PUMA ΔMLS, PUMA ΔBH3/ΔMLS), and fragment-based variants (PUMA N43, PUMA N44-102, PUMA N103-140). **H**, **I** Co-transfection of HEK293T cells with HA-tagged plasmids of various PUMA variants (as shown in **G**) and FLA**G**-tagged FASN plasmid was performed. After incubation with the HA antibody, Western blot analysis confirmed the interaction by detecting FLAG-FASN using the FLAG antibody (*n* = 6). Immunopurification results demonstrated the specific interactions between different variants of the PUMA plasmid and the FASN plasmid.

We further investigated PUMA’s molecular interactions related to lipid metabolism in ccRCC. Based on the IP and MS results, we overlapped three datasets: cytoplasmic IgG, cytoplasmic IP, and fatty acid biosynthesis. This analysis identified FASN, a key enzyme in fatty acid biosynthesis, as the only molecule interacting with PUMA (Fig. 3A). This finding aligns with Fig. S4G, suggesting that FASN is key to PUMA’s role in lipid metabolism. Endogenous and exogenous Co-IP experiments confirmed PUMA-FASN interaction in A-498, Caki-1, and HEK293T (Fig. 3B, C). In addition, Co-IP experiments in BAX/BAK DKO cells further validated this interaction, indicating that PUMA binds FASN independently of its pro-apoptotic function (Fig. S4H). Confocal imaging confirmed that FASN is primarily localized in the cytoplasm (Fig. 3D), and co-localizes with PUMA, particularly in the mitochondria region. These results highlight the essential role of FASN in PUMA-mediated lipid metabolism in ccRCC.

Next, we verified the PUMA-FASN interaction using FRET analysis and Co-IP assays. We constructed Clover2-PUMA and mRuby2-FASN plasmids, by labeling PUMA and FASN with a pair of FRET fluorophores Clover (donor) and mRuby2 (acceptor), respectively. FRET assay results confirmed a direct interaction between PUMA and FASN (Fig. 3E, F). We generated a series of PUMA deletion constructs (HA-PUMA ΔBH3, HA-PUMA ΔMLS, HA-PUMA ΔBH3/MLS, HA-PUMA-N43, HA-PUMA-N44–102, HA-PUMA-N103–140) and co-transfected them with FLAG-FASN plasmids into HEK293T cells (Fig. 3G). Co-IP results showed that N44-102 and N103-140 regions of PUMA are essential for its interaction with FASN (Fig. 3H, I). This differs from the previously recognized BH3 domain, which has been considered the primary interaction motif [20, 24]. A recent paper offered an alternative perspective, suggesting the binding domain of PUMA may extend beyond the traditional BH3-motif, and its subcellular localization may be influenced by its expression level and binding domains [40]. This aligns with our findings, which demonstrate that PUMA interacts with FASN outside of its BH3 domain, suggesting alternative binding sites involved in PUMA’s role in lipid metabolism. In conclusion, we found a protein-protein interaction between PUMA and FASN in ccRCC, with the N-terminal 44-140 sequence of PUMA playing a pivotal role in this interaction.

### PUMA mediates FASN expression to drive ccRCC progression and promote lipid accumulation

**Fig. 4:**
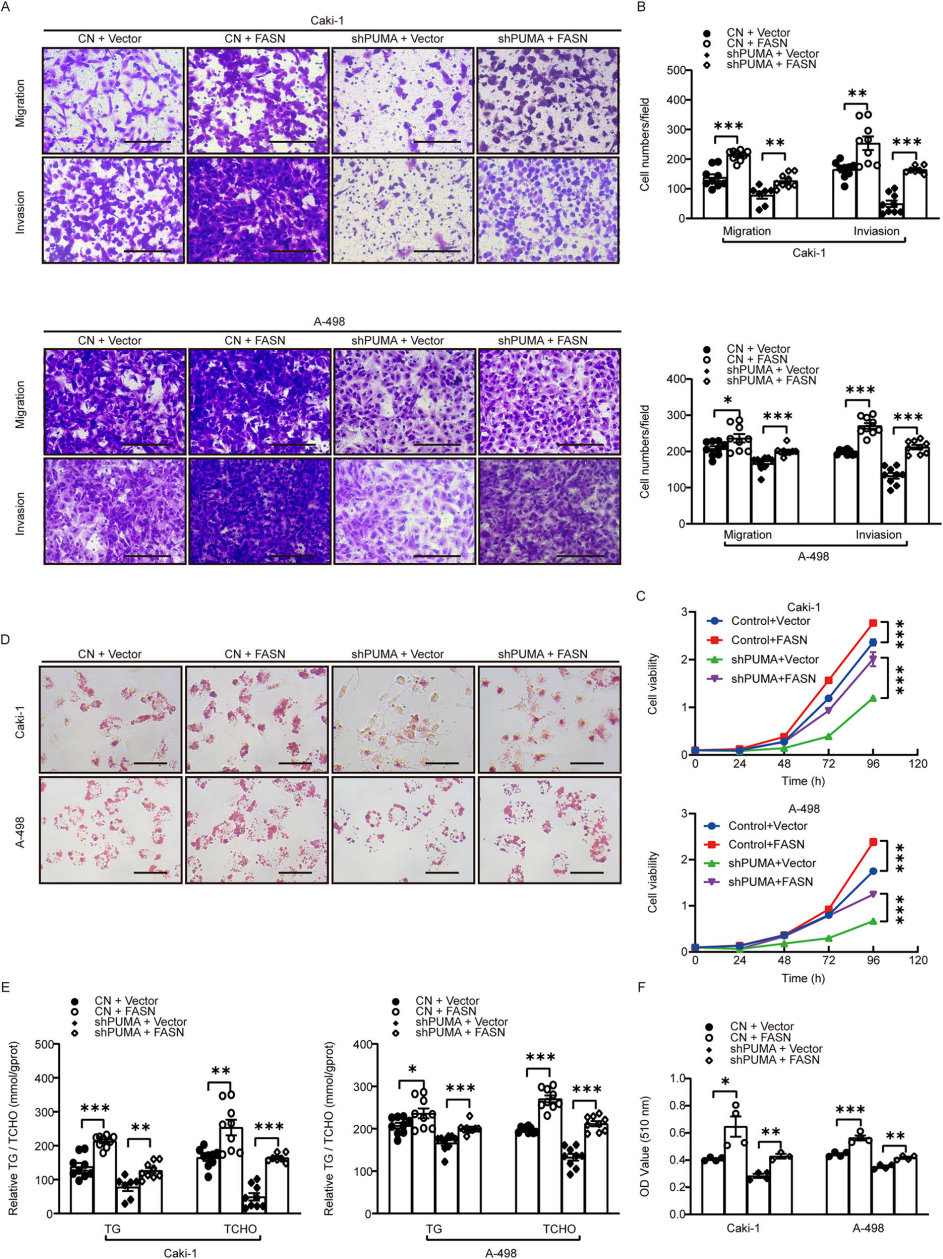
PUMA mediates FASN expression to drive ccRCC progression and promote lipid accumulation. Four distinct subgroups were established by transfecting vector plasmids (Vector) and FASN overexpression plasmids (FASN) in both the shPUMA group and the CN group. **A** Microscopic images from the transwell experiments depicting migration and invasion results in A-498 and Caki-1 cell lines for all four subgroups. Scale: 20 μm. **B** The statistical chart presents the outcomes of the transwell experiments shown in images (**A**) (*n* = 9). **C** The proliferation curve plot illustrates the cell viability in A-498 and Caki-1 cells for the four experimental groups, comparing the cell growth status across each group (*n* = 3). **D** ORO staining results for the four cell groups, captured at ×400 magnification in A-498 and Caki-1 cells. Scale: 20 μm. **E** and **F** The statistical data for lipid droplet content (*n* = 4), triglyceride, and total cholesterol content measurements in the four cell groups were depicted in histograms (*n* = 9).

FASN is crucial in fatty acid biosynthesis and has been associated with the tumorigenesis and progression of various cancers, including ccRCC [41]. We conducted transwell experiments to explore the regulatory relationship between PUMA and FASN and found that the supplementation of FASN almost restored the capabilities of migration and invasion in the PUMA knockdown group (Fig. 4A, B). Similar results were observed in cell viability assays (Fig. 4C). These findings suggest that PUMA regulates the oncogenic activity of FASN, thereby promoting tumor proliferation and migration in ccRCC. Similarly, ORO staining and measurements of triglycerides and cholesterol showed that FASN supplementation nearly restored lipid droplet formation and lipid metabolite levels in the PUMA knockdown group (Fig. 4D-F). These results are consistent with the transwell assay findings (Fig. 4A, B), further supporting the role of PUMA in promoting lipid metabolism and malignant progression in ccRCC via FASN.

To further investigate the functional relevance of the PUMA–FASN interaction, we overexpressed full-length PUMA and truncated constructs (N44–102 and N103–140) in PUMA knockdown cells. Transwell assays showed that PUMA supplementation restored cell migration, and the N44–102 constructs produced a similar effect (Fig. S5A, B). Cell proliferation and ORO staining assays reaffirmed that full-length PUMA restored proliferative capacity and lipid droplet levels, with the addition of the N44–102 motif achieving similar results (Fig. S5C–E). These results further underscore the critical role of the N44–102 motif of PUMA in directly interacting with FASN, thereby promoting cancer cell proliferation and lipid accumulation.

### PUMA enhances FASN stability and expression via ubiquitin-proteasome pathway

**Fig. 5:**
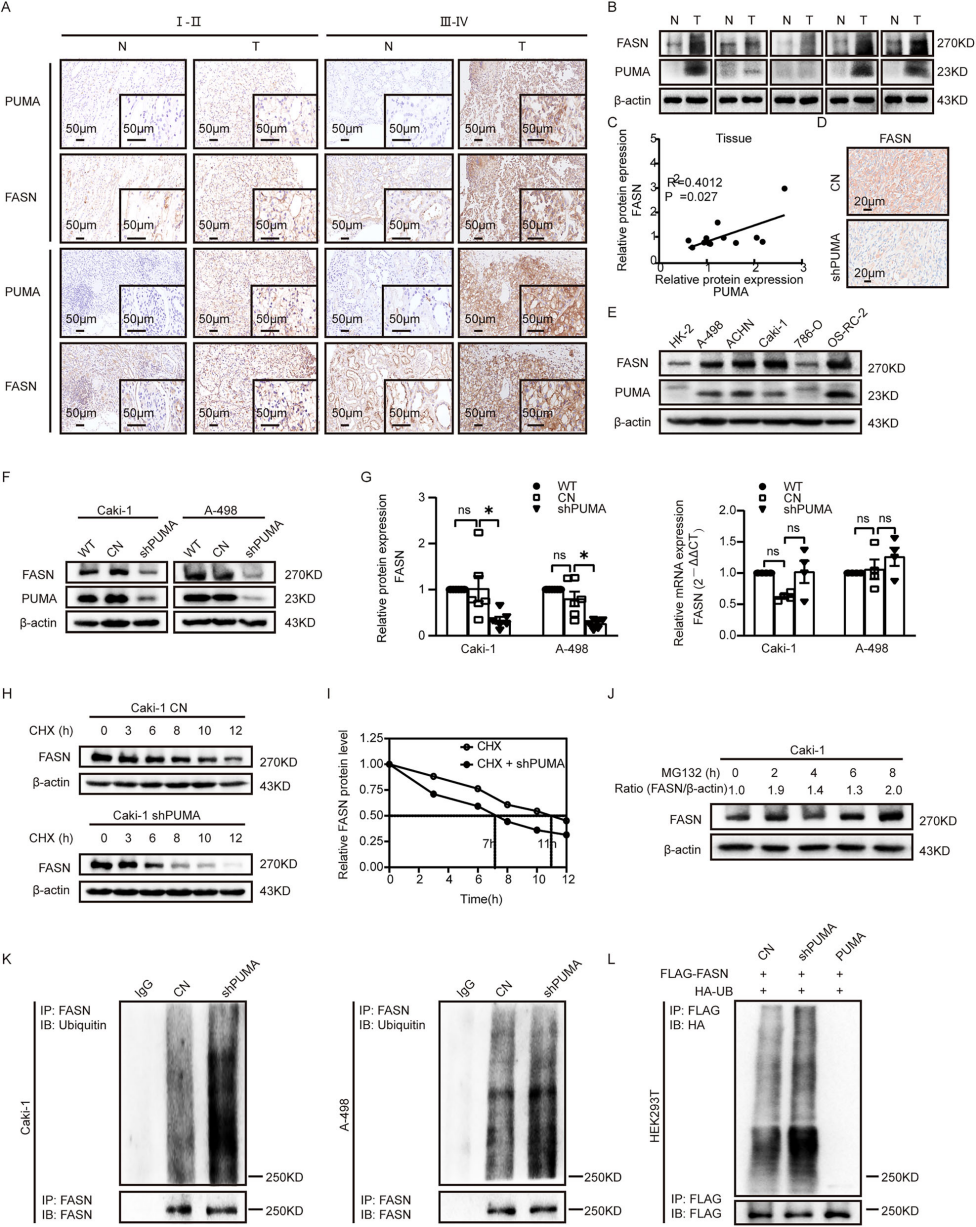
PUMA enhances FASN stability and expression via ubiquitin-proteasome pathway. **A** Immunohistochemistry microscopic images depict clinical tissue samples categorized into grades I–II and III–IV based on the degree of malignancy. N denotes normal tissue, and T denotes ccRCC tumor tissue. Staining antibodies used were PUMA and FASN, respectively. *n* = 10. Scale bar: 50 μm. **B** Protein expression of PUMA and FASN were assessed by western blot in 5 paired tissue specimens derived from ccRCC patients’ pathological tissues. T stood for ccRCC pathological tissues, while N referred to nearby healthy tissues. **C** A correlation analysis was performed on the protein expression of FASN and PUMA in primary tumor samples. Results: *R* = 0.6334, *R*^2^ = 0.4012, *P* = 0.027, *n* = 12. **D** Immunohistochemical analysis results of the expression of FASN, within subcutaneous tumor tissues from transplanted shPUMA and control ccRCC cell lines in nude mice. Scale: 20 μm. **E** The protein expression results of PUMA and FASN were assessed by Western blot across various renal cell lines, with β-actin serving as the cellular reference standard. **F** Western blot analysis on FASN protein expression in the shPUMA group, CN group, and wild type (WT) group. Separate analyses were conducted in the A-498 and Caki-1 cell lines. Additionally, statistical analyses (**G**) were performed for FASN protein (*n* = 6) and mRNA expression (*n* = 4). **H**, **I** FASN protein expression was assessed and quantitatively analyzed in both shPUMA and CN groups after treatment with CHX at different time points (*n* = 3). **J** FASN protein expression in Caki-1 cells was evaluated through western blot after exposure to MG132 (10 μM) at various time intervals (*n* = 3). **K** IP was performed in Caki-1 and A-498 cells with FASN antibodies to purify proteins from both the CN and shPUMA groups. To assess the ubiquitination levels of endogenous FASN, western blot analysis with a ubiquitin antibody was employed subsequently (*n* = 3). **L** In HEK293T cells, FLAG-tagged FASN plasmid, and HA-tagged UB plasmids were co-transfected, and after 48 h, FASN ubiquitination levels were examined in a western blot using HA antibodies following incubation with FLAG antibody (*n* = 3).

We explore further to reveal the mechanism of the interaction between PUMA and FASN. Immunohistochemical staining was performed on 10 pairs of clinical tissue samples based on various clinical stages (I–II and III–IV). The findings revealed an elevation in malignancy, accompanied by heightened staining for both PUMA and FASN (Fig. 5A). Importantly, a positive correlation was observed between PUMA and FASN staining intensities (see Fig. 5A). Subsequently, protein expression was examined in 12 pairs of ccRCC clinical tissue samples. A positive correlation was observed between the protein expression levels of FASN and PUMA, with FASN expression notably reduced in the PUMA knockdown group compared to the normal group (Fig. 5B, C). In vivo, FASN expression followed PUMA knock-down, showing a corresponding decrease (Fig. 5D). Expanding the investigation into ccRCC cell lines, Western blotting revealed a coordinated elevation in the protein levels of both FASN and PUMA (Fig. 5E). The protein and mRNA expression levels of FASN were highly elevated in ccRCC cell lines, similar to PUMA (Fig. S6A, B). Subsequent Western blot analysis indicated that the FASN protein expression decreased in the PUMA knockdown group, while it increased in the overexpressed PUMA cell lines, in contrast to the control group (Figs. 5F, G, and S6C, D). In qPCR analysis, no significant changes in FASN mRNA levels were observed, regardless of PUMA expression (Figs. 5G and S6D). These findings aligned with previous studies suggesting that FASN expression is primarily regulated at the post-translational level rather than at the mRNA level [42, 43]. Finally, compared to the control group, a protein stability assay demonstrated a shorter half-life of FASN in the PUMA knockdown group (Fig. 5H, I).

Recent studies have reported a correlation between apoptosis induction and lipid metabolism, suggesting that FASN overexpression may also be linked to apoptotic signaling [44]. To investigate whether PUMA regulates FASN independently of its pro-apoptotic activity, we assessed FASN protein levels in control and PUMA-overexpressing groups within BAX/BAK DKO cells. Western blot analysis showed that PUMA overexpression still led to increased FASN expression in the BAX/BAK DKO cells, consistent with observations in cells with intact BAX and BAK (Fig. S6E). These findings confirm that PUMA regulates FASN independently of its pro-apoptotic function.

To investigate the pathways involved in FASN protein degradation, we treated ccRCC cell lines with two classical protein degradation pathway inhibitors: MG132 (a ubiquitin-proteasome inhibitor, 10 μM), and chloroquine (an autophagy-lysosome inhibitor, 25 μM). The time gradient Western blotting showed that the MG132 treatment increased FASN protein levels (Fig. 5J and S6F, G). The PUMA knockdown group and the control group were treated with cycloheximide (CHX, a protein synthesis inhibitor, 25 μM). After 12 h, half of the cells in each group were treated with MG132, while the remaining half received no additional treatment. As depicted in Fig. S6H, the addition of MG132 resulted in an increase in FASN protein expression levels in both groups. To further investigate how PUMA regulates FASN, we performed ubiquitination IP assays, which showed increased FASN ubiquitination in the PUMA knockdown group compared to controls (Fig. 5K, L). We established a HEK293T cell line with stable PUMA knockdown, which was confirmed by Western blotting (Fig. S6I). In HEK293T cells, FASN ubiquitination levels were reduced with PUMA overexpression (Fig. 5L). These results support the conclusion that PUMA modulates FASN ubiquitination and regulates its protein stability via the ubiquitin–proteasome pathway.

### The PUMA–USP15–FASN axis and its synergistic effects with FASN inhibitors

**Fig. 6:**
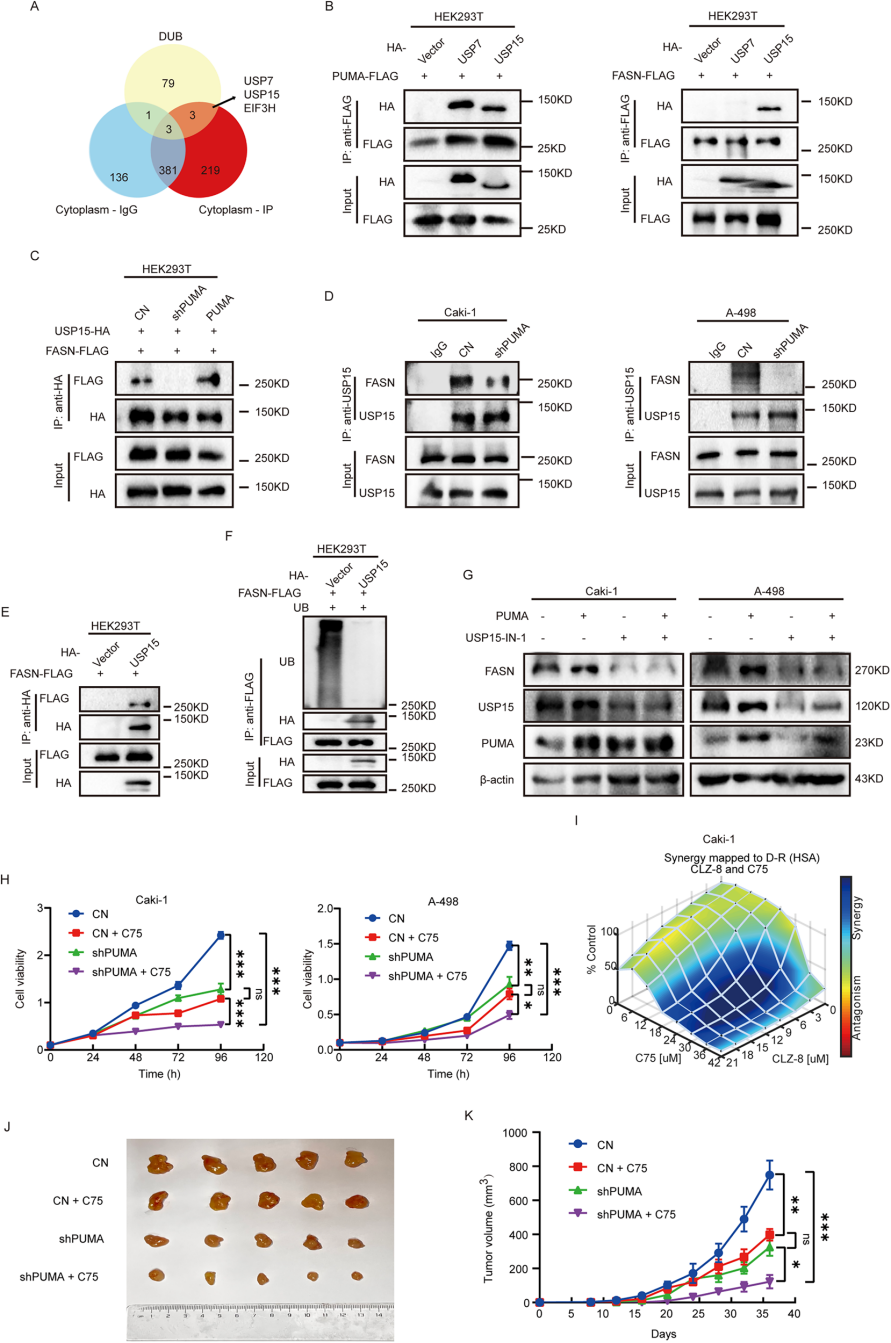
The PUMA–USP15–FASN axis and its synergistic effects with FASN inhibitors. **A** A Venn diagram illustrates the overlap between MS results and the deubiquitinase (DUB) data set. DUB data comes from the UbiBrowser 2.0 online database [60]. **B** HA-tagged deubiquitinates plasmid and FLAG-tagged PUMA or FASN plasmid were jointly transfected into HEK293T cells. After a 48-h incubation, FLAG-tagged proteins were purified via FLAG antibody IP, and western blot analysis was employed to examine the association of PUMA or FASN with deubiquitinates. **C** In HEK293T cells, HA-tagged USP15 plasmids and FLAG-tagged FASN plasmids were co-transfected into the CN group, shPUMA group, and PUMA group. USP15-HA protein was immunopurified using the HA tag antibody, and the extent of its interaction with FASN was determined through western blot analysis (*n* = 3). **D** IP was performed using USP15 antibody on the shPUMA and CN groups in Caki-1 and A-498 cells, and the interaction strength between endogenous USP15 and FASN was subsequently assessed through western blot analysis (*n* = 3). **E** USP15-HA and FASN-FLAG were overexpressed simultaneously in HEK293T cells with a 48-h incubation. USP15-HA protein was immunopurified using HA antibodies, and western blot analysis was then performed to examine its interaction with FASN (*n* = 3). **F** Simultaneous co-transfection of FASN-FLAG and UB was performed in HEK293T cells overexpressing USP15 and control cells. The subsequent purification of FASN-FLAG protein using FLAG antibody was followed by western blot analysis to assess its ubiquitination levels. **G** FASN protein expression in A-498 and Caki-1 cells was evaluated through western blot after exposure to USP15-IN-1 (3.76 μM) and overexpression of PUMA. **H** Both the shPUMA and CN group cells were treated with C75, a FASN inhibitor (35 μM). The cell viability in A-498 and Caki-1 cells was depicted in the proliferation curve plot, showcasing the differences among the groups (*n* = 3). **I** The three-dimensional surface plot (HAS model) visualizes the dose–response relationship of Caki-1 cells to different concentrations of CLZ-8 and C75 treatments. **J** The final set of images capturing subcutaneous tumor tissue in each group (*n* = 5) was documented, alongside the observation of tumor size every 4 days (**K**).

Since PUMA lacks a catalytically active domain, an intermediary protein is likely required to facilitate its regulation of FASN ubiquitination. Reviewing the previous results of MS analysis, we found that the deubiquitinases USP7 and USP15 might interact with PUMA (Fig. 6A). Nevertheless, the understanding and application of EIF3H as a deubiquitinating enzyme remain incomplete and require further comprehensive exploration [45]. In HEK293T cells, we conducted co-transfections involving the following combinations: (1) the plasmids FLAG-PUMA and HA-USP7, as well as FLAG-PUMA and HA-USP15; (2) FLAG-FASN and HA-USP7, as well as, FLAG-FASN and HA-USP15. Co-IP results indicated that USP15 was a regulatory adaptor between PUMA and FASN (Fig. 6B).

The PUMA-USP15-FASN axis was further validated by immunoprecipitation. In PUMA knockdown cells, the amount of FASN co-immunoprecipitated with USP15 was reduced compared to controls, while PUMA overexpression increased USP15-associated FASN levels (Fig. 6C). Similar results were observed in Caki-1 and A-498 cell lines, indicating a decrease in the level of FASN immunopurified by USP15 in the PUMA knockdown group (Fig. 6D). Furthermore, USP15 overexpression in HEK293T cells reduced the ubiquitination level of FASN, enhancing its immunopurification (Fig. 6E, F). The protein expression of FASN decreased significantly upon USP15 inhibition, independent of PUMA expression levels (Fig. 6G). Combining these results, we confirmed that PUMA regulates FASN ubiquitination via USP15, which consolidates the plausibility of the existence of the PUMA-USP15-FASN axis.

Previous findings have established PUMA as a promising therapeutic target for inhibiting the malignant progression of ccRCC. However, the adverse reactions and off-target effects of targeted drugs are dilemmas in the current treatment of ccRCC [6]. Given the limited clinical efficacy of FASN inhibitors alone, combining them with other targets is increasingly recommended [46]. Therefore, we delved into the combined efficacy of PUMA and FASN inhibitors. We evaluate the growth of tumor cell lines in four distinct experimental groups: the control group, the PUMA knockdown group, the control group treated with C75 (a FASN inhibitor, 35 μM), and the PUMA knockdown group treated with C75. Tumor cell proliferation was inhibited in the PUMA knockdown group and the control group treated with C75, with a more pronounced effect observed in the group receiving both C75 and PUMA knockdown (Fig. 6H). Specifically, the inhibition efficiency in the PUMA-KD group (compared to the control group) was significantly greater than that of the combined PUMA and FASN inhibition group (compared to the FASN inhibition group alone). CLZ-8 (a PUMA inhibitor) and C75 were used to assess their combined effects in A-498 and Caki-1 cells. Combenefit software was used to analyze the interaction between the two inhibitors [47]. The analysis using combenefit revealed a clear synergistic effect between CLZ-8 and C75 (Figs. 6I and S7A–C). Furthermore, dose–response analysis demonstrated that increasing CLZ-8 concentration allowed for a reduction in C75 dosage while maintaining efficacy (Fig. S7D, E). These findings underscore the synergistic potential of PUMA and FASN inhibition, suggesting their combined treatment as a promising therapeutic approach for ccRCC in clinical settings.

Therefore, we conducted additional experiments to verify the augmenting effect of knocking down PUMA on the inhibitor C75 in nude mice. The grouping was consistent with the in vitro experiment (Fig. 6H). The two groups treated with C75 (including the control group and the PUMA knockdown group in Fig. 6H) received intraperitoneal injection of 20 mg/kg every week, whereas the control group and the PUMA knockdown group without C75 received intraperitoneal injection of an equivalent volume of normal saline, totaling 36 days (Fig. S7F). The results are consistent with in vitro experiments (Fig. 6H). Tumor progression was reduced to a greater degree in the PUMA knockdown groups (Fig. 6J). The combined use of PUMA knockdown and C75 significantly slowed tumor growth compared to either single intervention (Fig. 6K). These findings suggest that PUMA functions within a broader regulatory network, with FASN serving as a key, though not exclusive, component. Additionally, the synergistic potential of PUMA and FASN inhibition supports their combined treatment as a promising therapeutic strategy for ccRCC in clinical settings.

### Mechanistic model of the PUMA–USP15–FASN axis in ccRCC

**Fig. 7:**
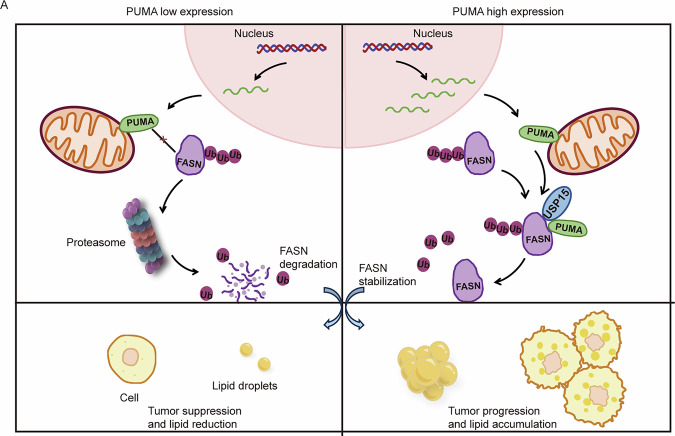
Mechanistic model of the PUMA-USP15-FASN axis in ccRCC. **A** Mechanism diagram: elevated PUMA expression in ccRCC leads to decreased FASN ubiquitination levels mediated by USP15, resulting in the stabilization of FASN protein expression. The molecular mechanism relationship axis of PUMA–USP15–FASN is delineated. Consequently, this induces increased lipid accumulation and facilitates advanced tumor progression, including proliferation, migration, and invasion.

In summary, we proposed a mechanistic model of the PUMA-USP15-FASN axis (Fig. 7A). This model revealed that abnormally high expression of PUMA regulates FASN in ccRCC, driving intracellular lipid accumulation and malignant progression of tumor. Our molecular studies reveal a novel regulatory mechanism in which PUMA binds to FASN via USP15 as an adapter, resulting in the formation of the PUMA–USP15–FASN axis. The establishment of the axis helped reveal the tumor progression and lipid metabolic reprogramming in ccRCC. Moreover, the combined regulation of PUMA-FASN inhibition can be more effectively translated into actual clinical applications, compared to individual interventions.

## Discussion

As a common malignant tumor of the urinary system, ccRCC is characterized not only by its resistance to traditional therapies but also by its unique metabolic demands. Recent studies indicate that systemic treatment of ccRCC is hampered by both drug tolerance and toxicity [6]. Traditional therapies primarily target the VHL pathway and its downstream effectors like mTOR and VEGF, yet these approaches frequently lead to resistance and poor prognosis [48]. This has steered current research towards metabolic reprogramming, with numerous metabolic targets showing therapeutic potential [49]. Our study contributes to this evolving understanding of the role of metabolic reprogramming, particularly through the identification of PUMA, a key downstream effector of p53, as a novel regulator of ccRCC progression.

Although widely recognized for its pro-apoptotic role in various cancers, PUMA exhibits a paradoxical function in ccRCC. Our study uniquely reveals that PUMA is highly expressed in ccRCC tissues and cell lines, correlating with advanced tumor stages and poor prognosis. Notably, our findings confirm that PUMA is not correlated with apoptosis in ccRCC (Fig. S1), contrasting with its established role in other cancers [25, 50]. This disconnection prompts us to investigate alternative functions of PUMA in ccRCC.

Our data reveal that PUMA’s role in ccRCC is closely linked to lipid metabolism. Mass spectrometry analysis indicated that PUMA interacts with key metabolic enzymes, notably FASN. As a crucial enzyme in de novo fatty acid biosynthesis, FASN is overexpressed in various malignancies, including ccRCC [49, 51–53]. Its protein levels are pivotal for its lipid-synthesizing function. The N44-102 sequence of PUMA is crucial for binding to FASN and promoting lipid droplet accumulation and tumor progression. Given that FASN is regulated by post-translational modifications such as ubiquitination [54], we identified USP15 as a significant regulator of FASN in ccRCC, aligning with previous findings that other USPs, such as USP2a, USP13, and USP38, modulate FASN stability [55–57]. Notably, we demonstrated that the combination of PUMA knockdown and C75, an early clinical FASN inhibitor [58], enhanced the inhibitory effect on ccRCC growth compared to individual treatments, in both in vitro and in vivo experiments. These findings suggest that the combined administration of targeted PUMA and FASN inhibitors is a promising therapeutic strategy, validating our mechanistic insights and offering a potential path forward in ccRCC treatment.

These promising findings pave the way for further exploration into the regulation of PUMA. First, while it is known that PUMA is a crucial downstream effector of *p53*, our study reveals that it upregulated in ccRCC, promoting tumor progression, without a significant correlation to cell apoptosis. Beyond *p53*, PUMA is regulated by transcription factors such as NF-κB, IRF-1, and FoxO3a, and negatively by Slug and p73 [22]. Future studies using CRISPR/Cas9 technology [59] to identify upstream regulatory factors could clarify PUMA’s regulatory network. Second, our study suggests that PUMA’s function in ccRCC is influenced not only by expression levels but also by its interaction with effector proteins like FASN. While our study focused on fatty acid biosynthesis and lipid droplet accumulation, other metabolic pathways remain to be explored. Our results suggest potential associations with the TCA cycle, highlighting the need for further investigation into other metabolic processes, such as glycolysis and amino acid metabolism. Lastly, our study does not provide a detailed analysis of the spatial dynamics of PUMA–FASN interactions. While PUMA and FASN were observed to co-localize in the cytoplasm, primarily in mitochondria, their precise spatial relationship remains to be explored. A recent study suggests that the mitochondrial localization of PUMA is dynamically adjusted by protein expression levels and its interacting partners [40]. This implies that PUMA–FASN interactions may vary depending on the spatial and temporal context within the cell. The N44-102 sequence of PUMA appears to influence its role in ccRCC progression and lipid synthesis, suggesting this region may affect its localization and function. However, further investigation is needed to fully elucidate the mechanisms regulating PUMA’s shift from a pro-apoptotic protein to a metabolic roles. Future studies using high-resolution imaging techniques and complementary experimental approaches could provide valuable insights into how PUMA–FASN interactions evolve under different cellular contexts and stress conditions.

In conclusion, our study reveals that high PUMA expression promotes tumor proliferation, migration, invasion, and lipid accumulation in ccRCC. By interacting with FASN and USP15, PUMA drives metabolic reprogramming and tumor growth. This pathway challenges the conventional view of PUMA as solely pro-apoptotic and provides new insights into cancer biology. Targeting PUMA, particularly in combination with FASN inhibition, presents a promising therapeutic strategy to enhance ccRCC treatment.

## Supplementary information

Supplementary figure

Supplementary table

Original western blot

## Data Availability

To protect patient privacy, human clinical data from this study are not publicly available but can be obtained from the authors upon request. Protein sequences and original protein sequence data generated in this study are also available upon request. Full Western blot images and additional supporting data are available in the Supplementary Material.
