# Supplementary material for: PUMA reduces FASN ubiquitination to promote lipid accumulation and tumor progression in human clear cell renal cell carcinoma
Source: Cell Death Dis. 2025 Jun 19;16(1):460. doi: 10.1038/s41419-025-07782-y (PMC12177072; doi:10.1038/s41419-025-07782-y)
Supplement: Supplementary file 1 — Supplementary figure [file 41419_2025_7782_MOESM1_ESM.pdf]

Supplementary Figure S1

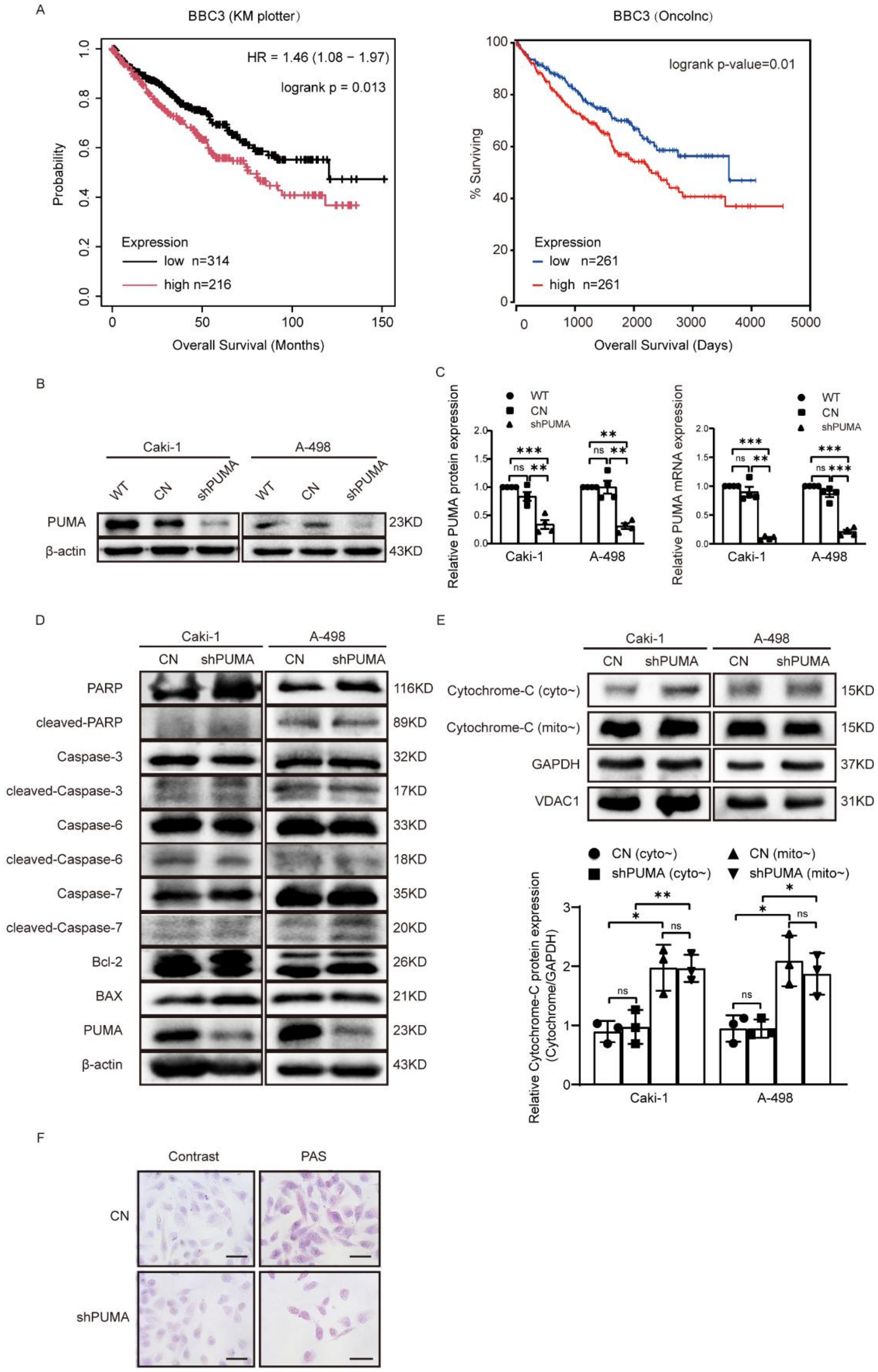

Fig. S1. PUMA demonstrates no significant correlation with either cell apoptosis or glycogen accumulation. **(A)** Kaplan-Meier survival analysis of ccRCC patients (n=530) comparing high and low PUMA expression, using the best cutoff from the kmplotter website and the median value from the Oncolnc website (n=532). **(B)** Western blot analysis results showing protein expression in the shPUMA group, WT, and CN group in A-498 and Caki-1 cells. Statistical evaluation of PUMA downregulation in both protein and mRNA is shown in **(C)** (n=4). Western blot analysis of whole-cell **(D)** and cytoplasmic/mitochondrial **(E)** protein expression of apoptosis markers in both the CN and shPUMA groups of Caki-1 and A-498 cell lines (n=3). **(F)** Micrographs illustrating the outcomes of PAS staining conducted on cells from both the CN and shPUMA groups. Scale bar: 10 μm.

Supplementary Figure S2

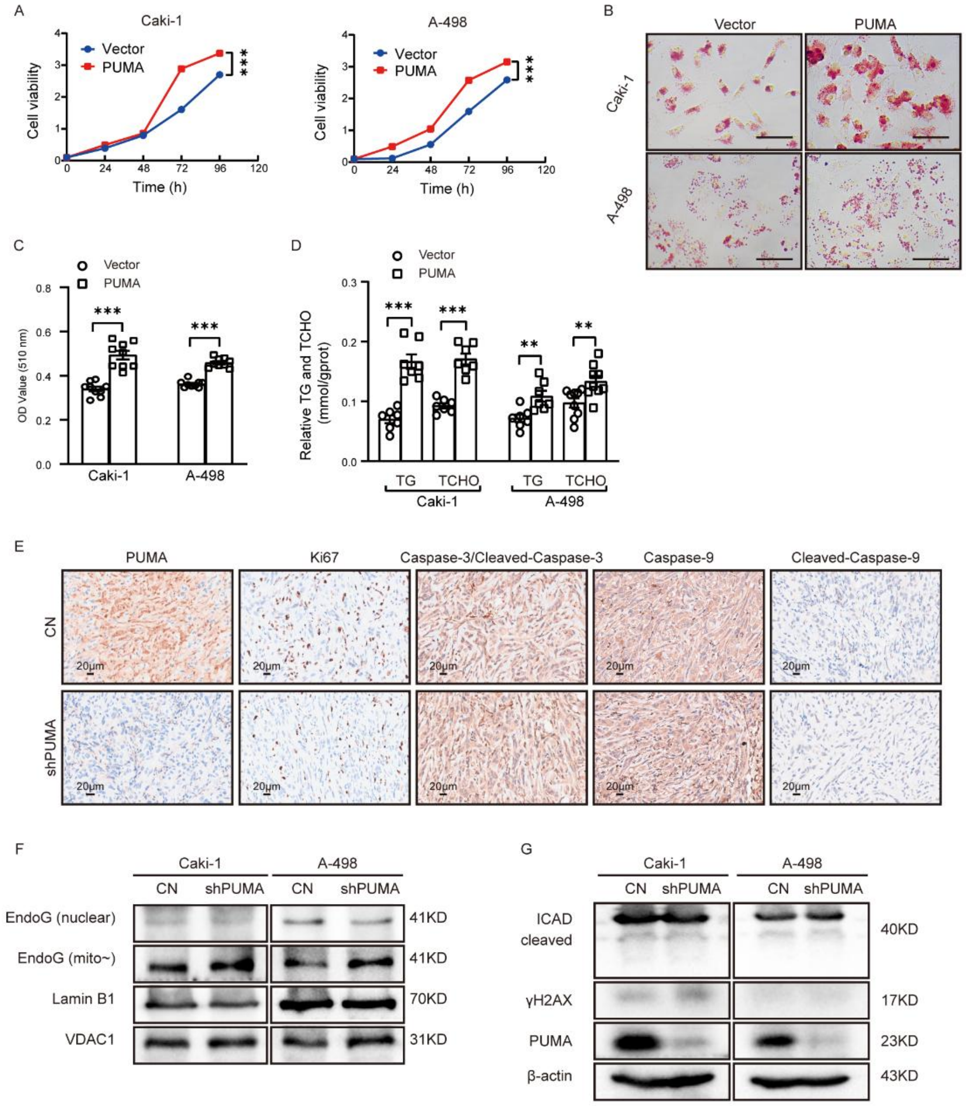

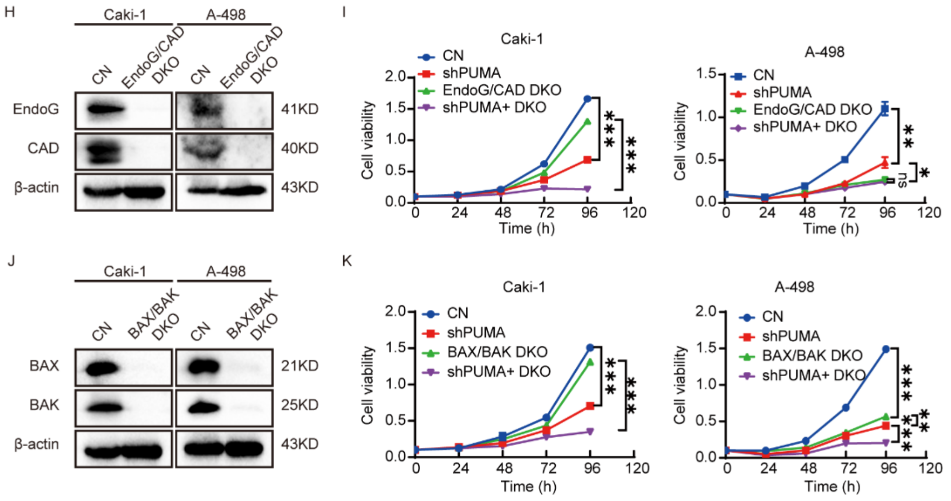

Fig. S2. PUMA's role in cancer promotion independent of apoptosis. **(A)** Comparison of cell proliferation curves between the PUMA overexpression group (PUMA) and the control group (Vector) in A-498 and Caki-1 cells (n=3). **(B)** Oil Red Staining results showing lipid droplets in the PUMA overexpression group (PUMA) and the control group (Vector) (n=8). Scale bar is 20 μm. **(C)** Bar graphs present statistical analysis, comparing cellular lipid droplet content between the PUMA and Vector groups. The comparison of TG content and TCHO content is shown in **(D)**. **(E)** Immunohistochemical analysis results of the expression of PUMA and apoptotic markers, within subcutaneous tumor tissues from the shPUMA group and the CN group. Antibodies included the malignancy marker Ki67, and the apoptotic markers Caspase-3 and-9, scale: 20μm. **(F)** Western blot analysis of EndoG in mitochondria (mito~) and nuclear fractions of A-498 and Caki-1 cells, with VDAC1 as a mitochondrial marker and Lamin B1 as a nuclear fraction. n=3. **(G)** Western blot analysis of ICAD and γH2AX in A-498 and Caki-1 cells. n=3. **(H)** Western blot analysis of EndoG and CAD in EndoG/CAD DKO cells. **(I)** Comparison of cell proliferation between shPUMA and control groups in EndoG/CAD DKO cells. **(J)** Western blot analysis of BAX and BAK in BAX/BAK DKO cells. **(K)** Comparison of cell proliferation between shPUMA and control groups in BAX/BAK DKO cells.

Supplementary Figure S3

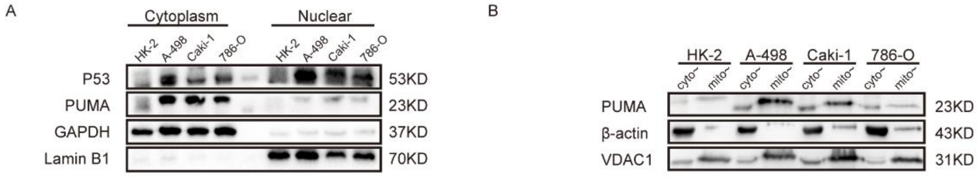

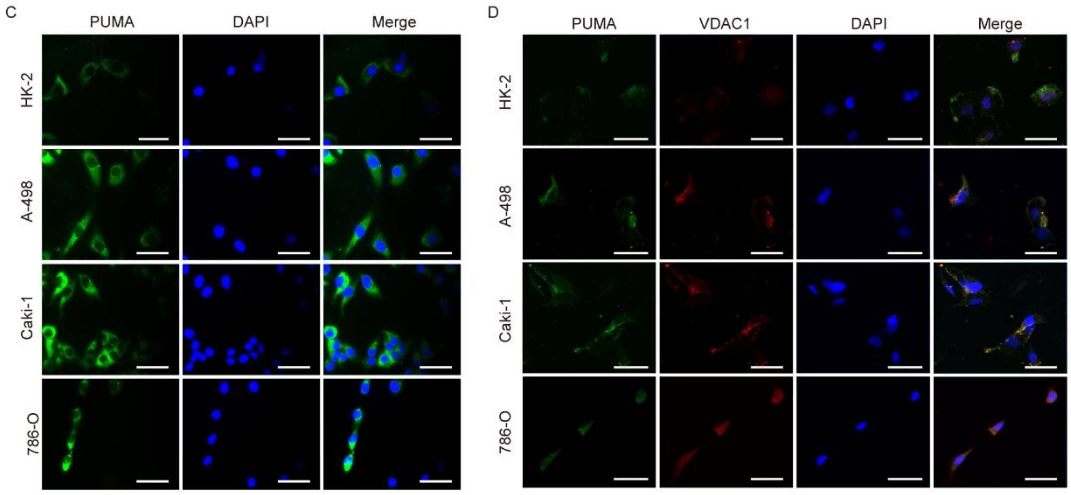

Fig. S3. PUMA localizes in the cytoplasm and primarily in the mitochondria. **(A)** PUMA protein expression was assessed by Western blot analysis in both cytoplasmic and nuclear fractions of HK-2, A-498, Caki-1, and 786-O cells. Standards include β-actin for whole-cell, GAPDH for cytoplasmic, and Lamin B1 for nuclear fractions (n=3). **(B)** The protein expression of PUMA was analyzed by Western blot in mitochondria (mito~) and cytoplasm (cyto~) of HK-2, A-498, Caki-1, and 786-O cells. VDAC1 was used as the mitochondrial reference protein (n=3). **(C)** Immunofluorescence images of PUMA staining in HK-2, A-498, Caki-1, and 786-O cells, utilizing a PUMA antibody (green) and DAPI (blue). Merged images illustrate the overlay of the two fluorescent channels. Scale: 5μm. **(D)** Immunofluorescence results depict PUMA staining (green) alongside VDAC1 (red, mitochondrial marker protein). The merged images illustrate the overlay of two fluorescence channels with the images having a scale of 5μm.

Supplementary Figure S4

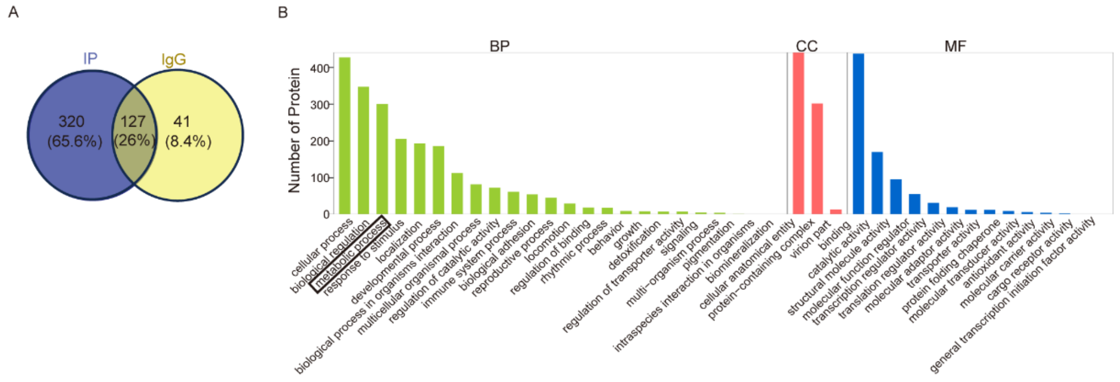

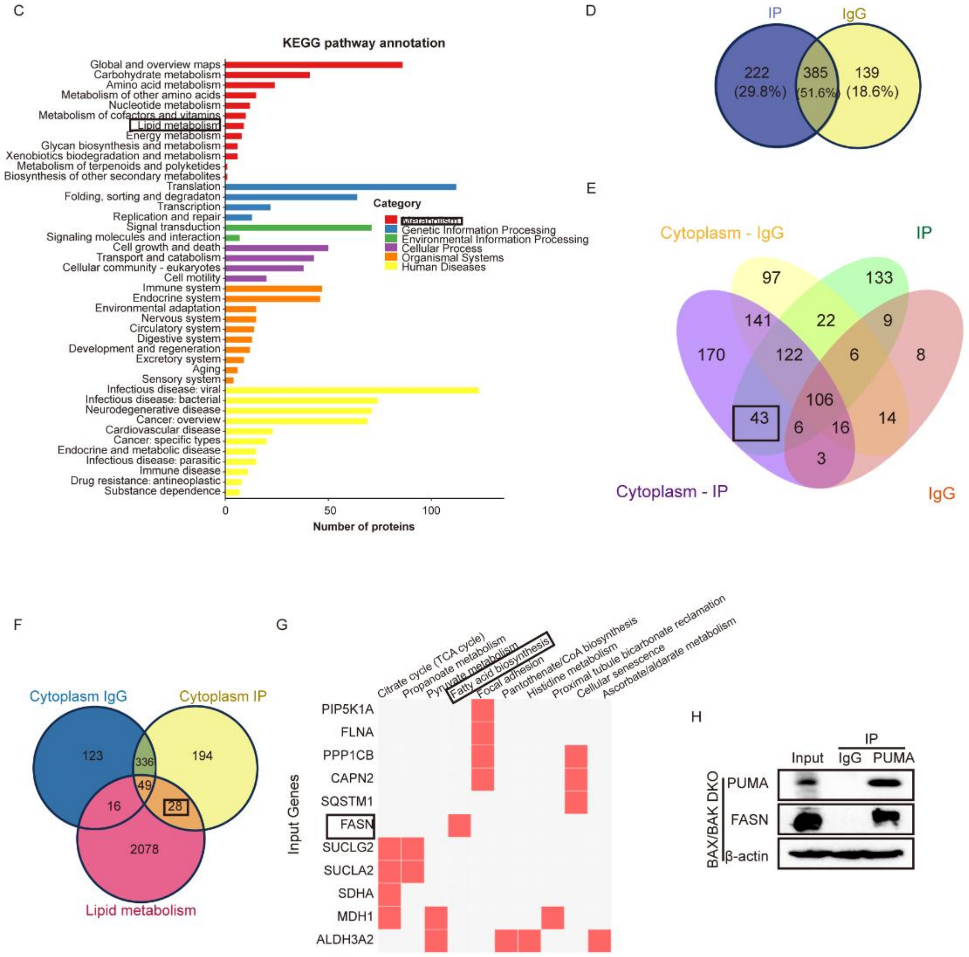

Fig. S4. FASN as a key molecule in PUMA-mediated fatty acid biosynthesis. **(A)** A Venn diagram depicts the intersection results between the whole-cell IgG and whole-cell IP group datasets. **(B)** The functional analysis of proteins immunoprecipitated by the PUMA antibody shows their association with specific biological processes (BP), cellular components (CC), and molecular functions (MF). **(C)** Findings from KEGG pathway annotation of protein profiling. Present the protein count in each group categorized by different functional classes. **(D)** A Venn diagram depicts the intersection results between the cytoplasm IgG (P'IgG), and IP (P'IP) group datasets. **(E)** Intersection analysis of the whole-cell IgG, whole-cell IP, P'IgG, and P'IP datasets reveals 43 proteins common to both the whole-cell IP and P'IP groups. **(F)** A Venn diagram depicts the intersection results among the P'IgG, P'IP, and lipid metabolism dataset. The intersection analysis reveals 28 proteins common to both the cytoplasm IP group and the lipid metabolism dataset. **(G)** The outcomes of the 28 proteins were submitted to the Enrichr website. The figure illustrates the top 10 enriched terms results based on the combined score,

emphasizing the fatty acid biosynthesis pathway and the corresponding FASN protein with a higher combined score. **(H)** IP results demonstrate the interaction between endogenous PUMA and FASN in BAX/BAK DKO cells (n=3).

Supplementary Figure S5

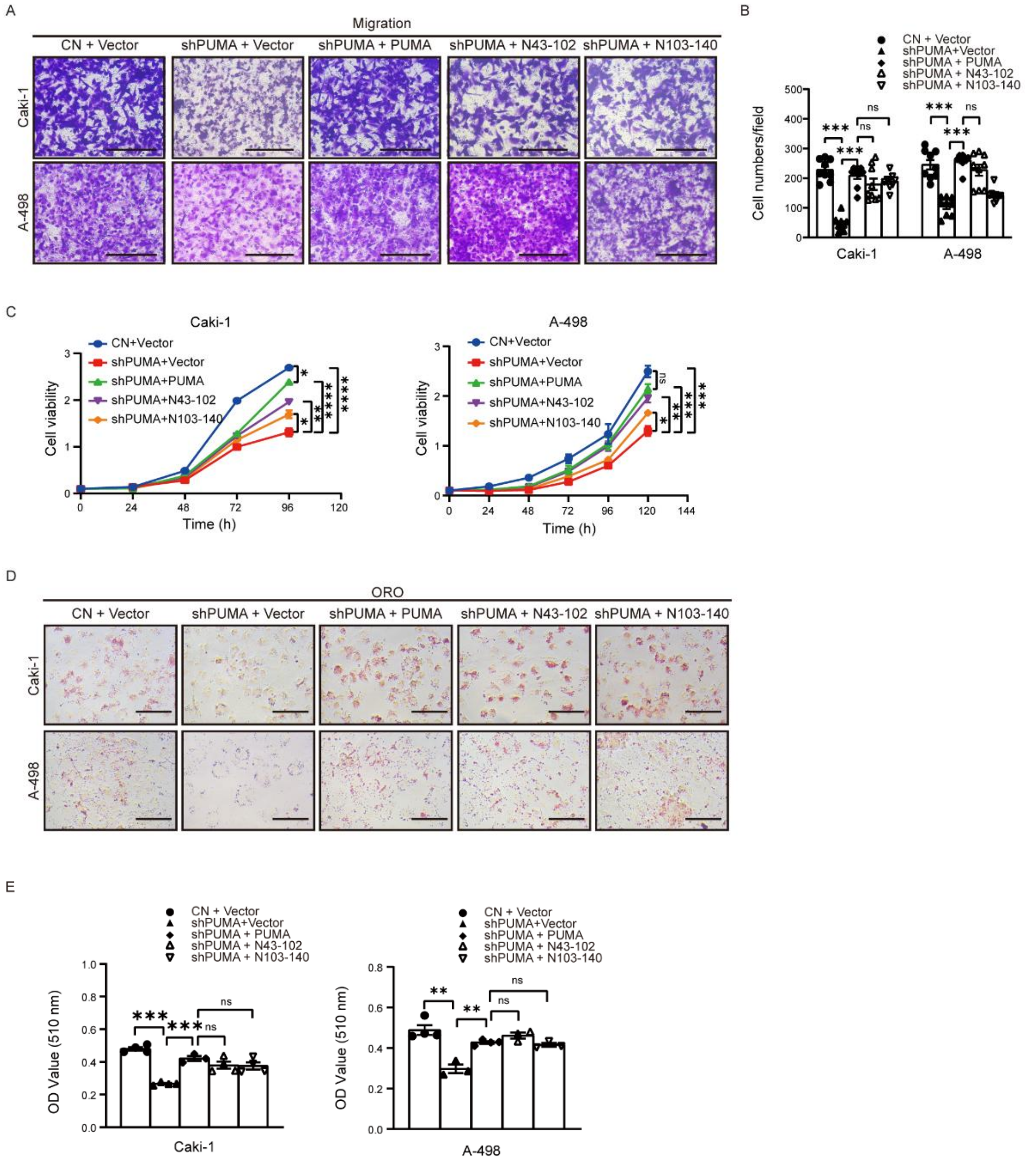

Fig. S5. The N44-102 motif of PUMA is the key sequence promoting cancer cell proliferation and lipid accumulation. **(A)** Microscopic images from the transwell experiments depicting migration results in A-498 and Caki-1 cell lines for all groups. Scale: 20 μm. **(B)** The statistical chart presents the outcomes of the transwell experiments shown in images **(A)**. **(C)** The proliferation curve graph compares the

cell growth status in A-498 and Caki-1 cells among the five groups. **(D)** The ORO staining results for five cell groups were captured at 400 × magnification in both A-498 and Caki-1 cells. Scale: 20 μm. **(E)** Statistical data for lipid droplet content measurements in the five cell groups are depicted in histograms.

Supplementary Figure S6

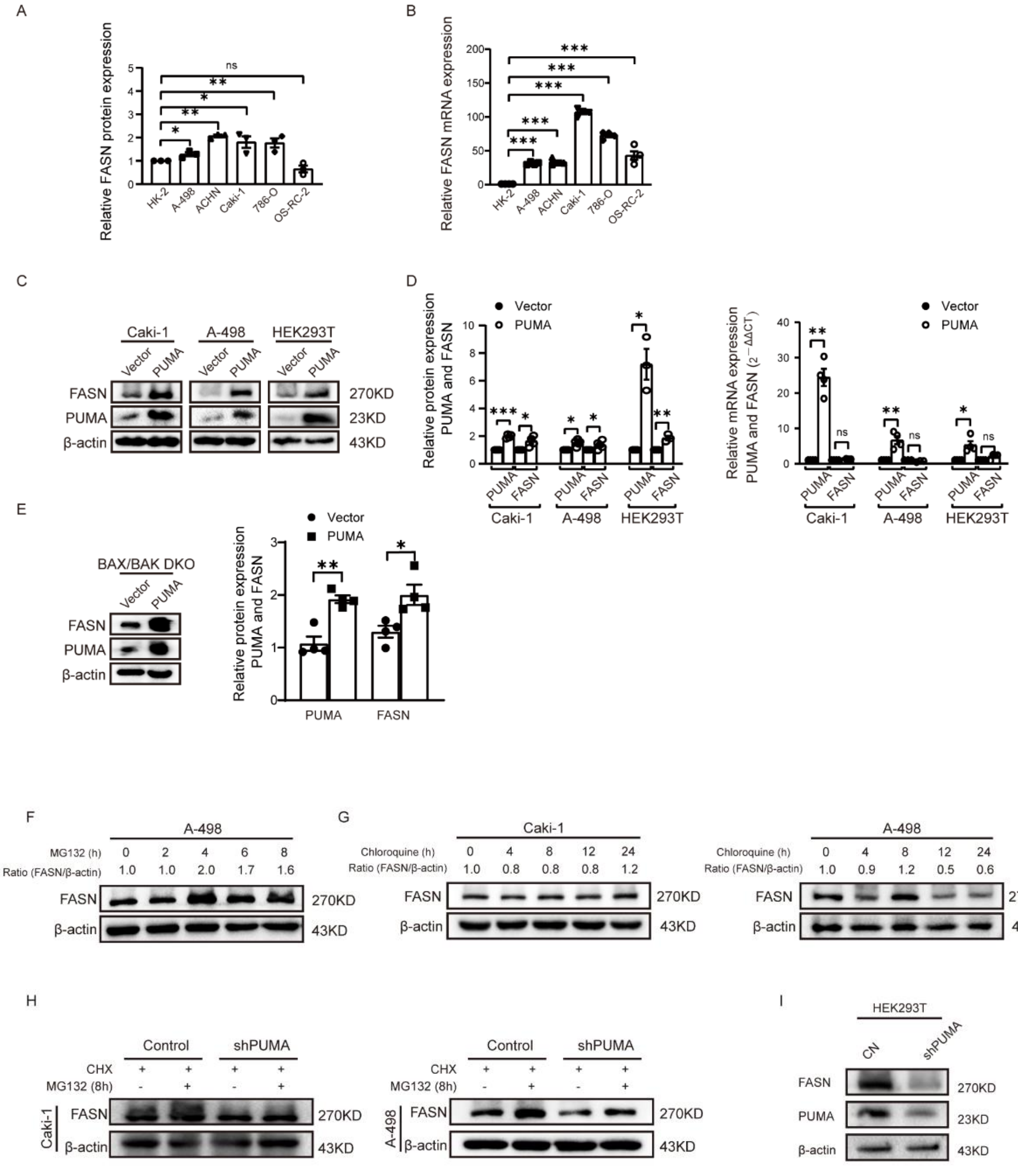

Fig. S6. PUMA enhances FASN protein stability via the ubiquitin-proteasome pathway. **(A)** A histogram visually represents the statistical analysis of relative FASN expression in both protein and mRNA **(B)** across various renal cell lines. **(C)** FASN protein expression was measured using Western blot in A-498, Caki-1, and

HEK293T cells after 48 hours of transfection with PUMA overexpression plasmid and Vector (n=3). **(D)** Histograms presenting the statistical analysis of FASN protein and mRNA expression in A-498, Caki-1, and HEK293T cells after overexpression of PUMA. **(E)** Western blot analysis on FASN protein expression in the PUMA-overexpression with BAX/BAK-DKO group, CN group. Additionally, statistical analysis was performed for FASN protein (n=3). **(F)** FASN protein expression in A-498 cells was evaluated through Western blot after exposure to MG132 (10 μM) at various time intervals (n=3). **(G)** Western blot analyses were conducted to assess the FASN protein expression after chloroquine (25 μM) treatment in Caki-1 and A-498 cells over various time points (n=3). **(H)** We pre-treated cells with CHX (25 μM) for a period of 12 hours in both CN and shPUMA groups of Caki-1 and A-498 cells. Subsequently, they were exposed to either DMSO or MG132 (10 μM) for 8 hours. The assessment of FASN protein expression was performed through Western blot analysis. **(I)** Western blot analysis results of shPUMA group and the CN group protein expression in HEK293T cells.

Supplementary Figure S7

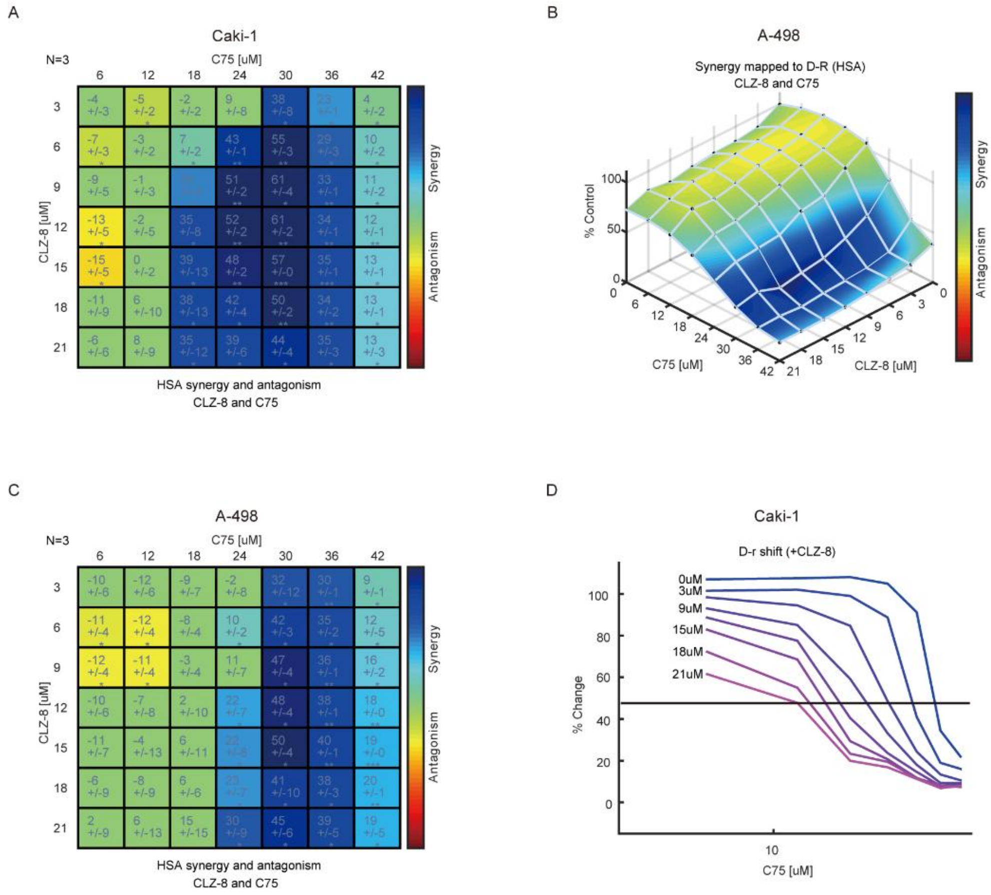

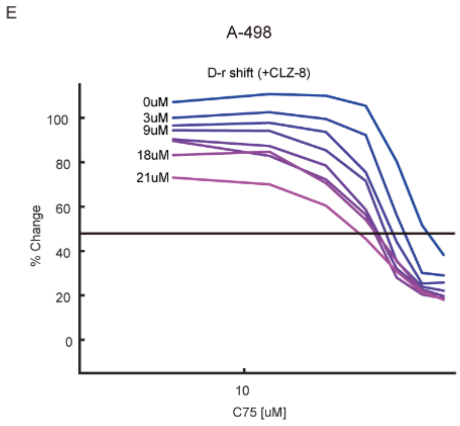

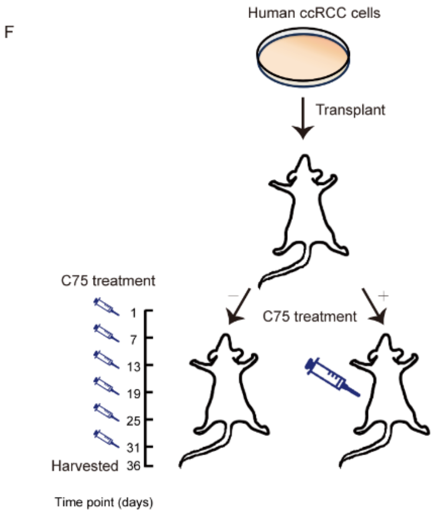

Fig. S7. The combination of PUMA and FASN inhibitors creates a synergistic effect. **(A)** The analysis table of the dose-synergy relationship for the CLZ-8-C75 combination in Caki-1 cells. **(B)** The three-dimensional surface plot (HAS model) illustrates the dose-response relationship of A-498 cells under various concentrations of CLZ-8 and C75. Table **(C)** presents the analysis of the dose synergy relationship for the drug combinations. **(D)** The dose-response inhibitory efficiency curves for CAKI-1 and A-498 **(E)** cells treated with CLZ-8 and C75 at various concentration gradients. **(F)** Schematic diagram of animal experiments. Human-derived ccRCC cells were subcutaneously transplanted into nude mice and subsequently divided into a C75 administration group and a control group. The former was injected intraperitoneally every week.
