# Supplementary material for: PUMA reduces FASN ubiquitination to promote lipid accumulation and tumor progression in human clear cell renal cell carcinoma
Source: Cell Death Dis. 2025 Jun 19;16(1):460. doi: 10.1038/s41419-025-07782-y (PMC12177072; doi:10.1038/s41419-025-07782-y)
Supplement: Supplementary file 2 — Supplementary table [file 41419_2025_7782_MOESM2_ESM.pdf]

Supplementary Tables

Table S1. Antibody information
Table S2. Primer sequences
Table S3. List of abbreviations

Table S1

p53 Rabbit pAb (A3185, ABclonal)
PUMA α/β (G-3) (sc-374223, Santa Cruz Biotechnology)
PUMA α (B-6) (sc-374223, Santa Cruz Biotechnology)
PUMA Rabbit mAb (D30C10, Cell Signaling Technology)
Fatty Acid Synthase Rabbit mAb (C20G5, Cell Signaling Technology)
HA-Tag Rabbit mAb (C29F4, Cell Signaling Technology)
Mouse anti HA-Tag mAb (AE008, ABclonal)
Mouse anti DDDDK-Tag mAb (AE005, ABclonal)
USP15 Polyclonal antibody (14354-1AP, Proteintech)
ubiquitin antibody (10201-2-AP, Proteintech)
β-Actin Rabbit mAb (AC026, ABclonal)
Lamin B1 Rabbit mAb (A11495, ABclonal)
VDAC1 / Porin Rabbit pAb (A15735, ABclonal)
GAPDH Mouse mAb (AC002, ABclonal)
Caspase-3/Cleaved-Caspase-3 Rabbit mAb (A19654, Abclonal)
Caspase-6 Rabbit mAb (A19665, ABclonal)
Cleaved-Caspase-6 Rabbit pAb (A23061, ABclonal)
Caspase-7 p12 Rabbit mAb (A19666, ABclonal)
Caspase-7/Cleaved-Caspase-7 Polyclonal antibody (27155-1-AP, Proteintech)
ENDOGL1 / ENGL Rabbit mAb (A20881, ABclonal)
CAD Antibody (sc-374067,Santa Cruz Biotechnology)
DFFA Rabbit pAb (A12431, ABclonal)
Phospho-Histone H2AX-S139 Rabbit mAb (AP1555, ABclonal)
PARP Rabbit mAb (F0148, Selleck)
Cleaved PARP Rabbit mAb (F0136, Selleck)
Cytochrome C Mouse mAb (F0137, Selleck)
Bax Antibody (sc-70408, Santa Cruz Biotechnology)
BAK1 Rabbit pAb (A21283,ABclonal)
Ki67 Rabbit mAb (A20018, ABclonal)
DAPI (C1002, Beyotime)
Mito-Tracker Red CMXRos (C1049B, Beyotime)
ABflo® 488-conjugated Goat anti-Mouse IgG (H+L) (AS037, ABclonal)
Alexa Fluor 546 Donkey Anti Mouse IgG (H+L) (ANT026, AntGene)
FITC-labeled Anti-Mouse (A0568, Beyotime)
Cy3-labeled Anti-Rabbit (A0516, Beyotime)

Table S2

Primer	Sequence
β-actin	
Forward	5' CATGTACGTTGCTATCCAGGC 3'
Reverse	5' CTCCTTAATGTCACGCACGAT 3'
PUMA	
Forward	5' GACCTCAACGCACAGTACGAG 3'
Reverse	5' AGGAGTCCCATGATGAGATTGT 3'
FASN	
Forward	5' AAGGACCTGTCTAGGTTTGATGC 3'
Reverse	5' TGGCTTCATAGGTGACTTCCA 3'

Table S3

Full Name	Abbreviation
Kidney clear cell carcinoma	KIRC
Kidney papillary cell carcinoma	KIRP
Colon cancer	COAD
Large B-cell lymphoma	DLBC
Esophageal cancer	ESCA
Head and neck cancer	HNSC
Pancreatic cancer	PAAD
Rectal cancer	READ
Stomach cancer	STAD
Thyroid cancer	THCA
Thymoma	THYM
Mitochondrial localization signal	MLS
BH3-only protein natural born killer	Nbk
Bcl-2-interacting killer	Bik
Bcl-2-associated X protein	BAX
Bcl-2 antagonist killer	BAK
Tricarboxylic acid cycle	TCA cycle
