# Supplementary material for: PUMA reduces FASN ubiquitination to promote lipid accumulation and tumor progression in human clear cell renal cell carcinoma
Source: Cell Death Dis. 2025 Jun 19;16(1):460. doi: 10.1038/s41419-025-07782-y (PMC12177072; doi:10.1038/s41419-025-07782-y)

Original Western blot

Fig.1 (K)

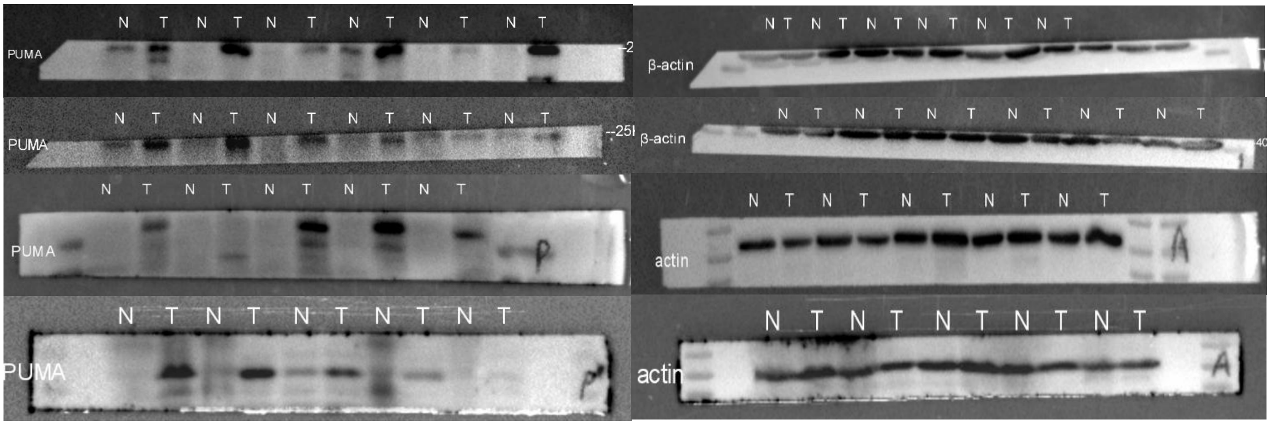

Fig.1 (M)

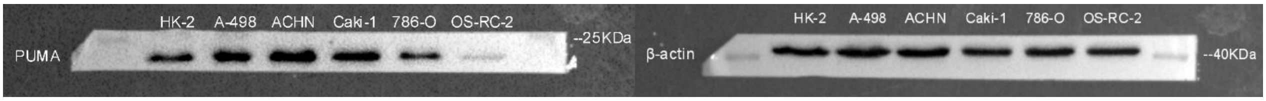

Fig.3 (B)

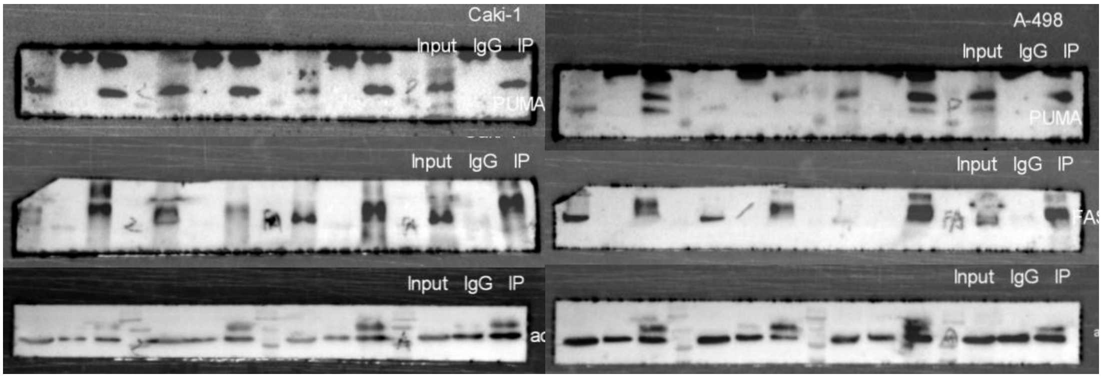

Fig.3 (C)

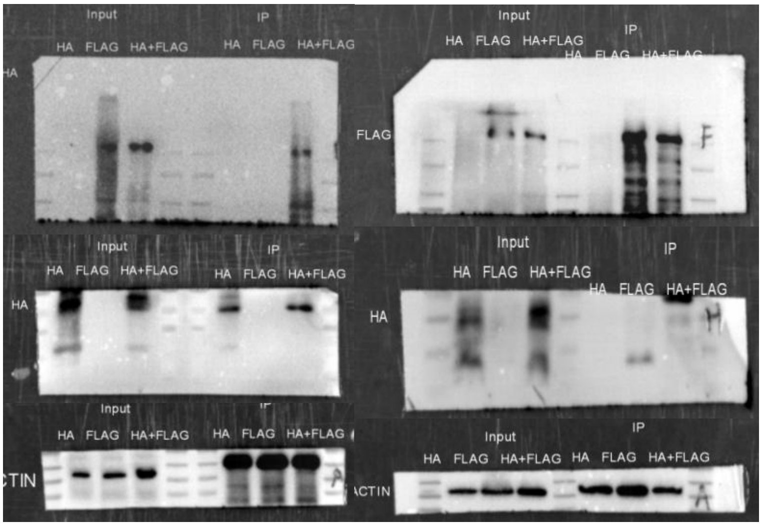

Fig.3 (H)

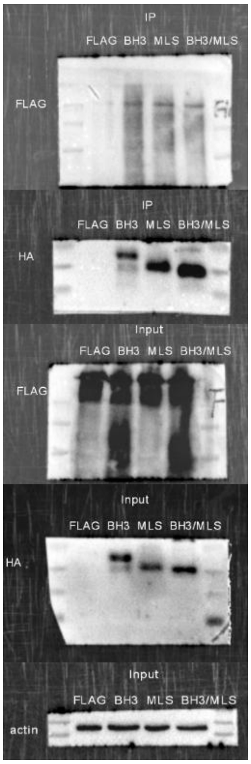

Fig.3 (I)

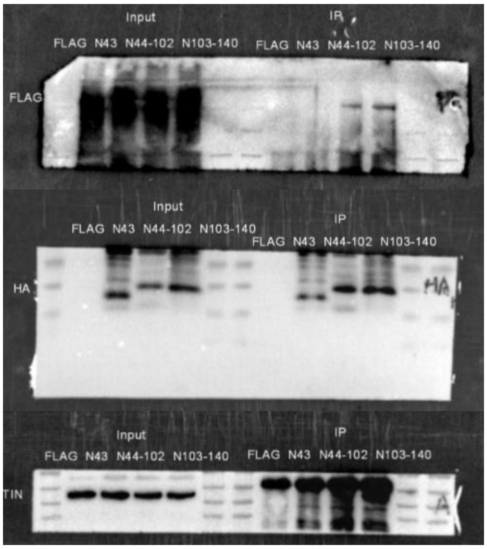

Fig.5 (B)

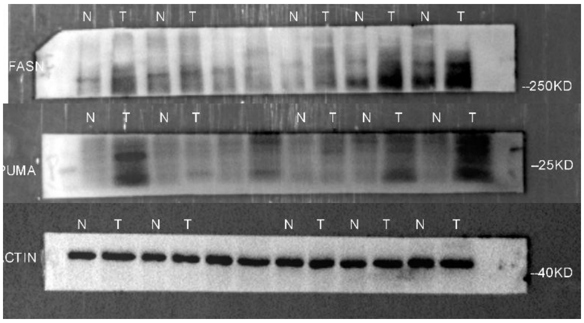

Fig.5 (E)

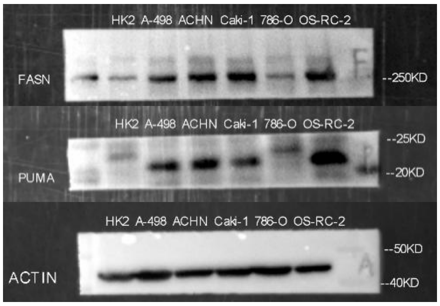

Fig.5 (F)

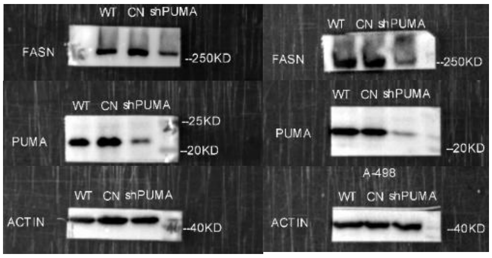

Fig.5 (H)

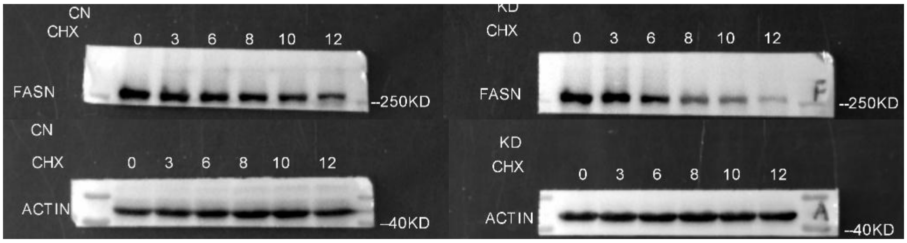

Fig.5 (J)

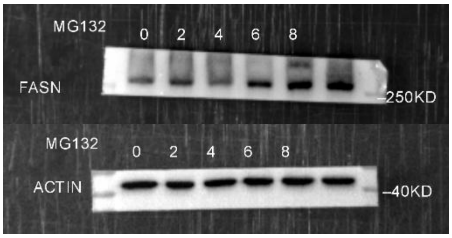

Fig.5 (K)

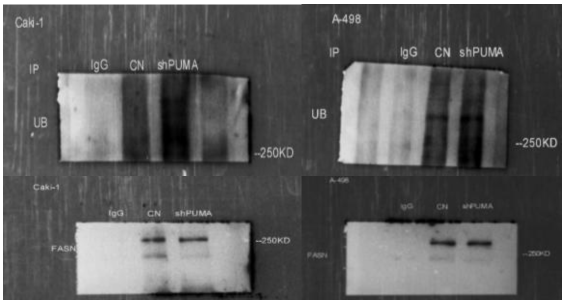

Fig.5 (L)

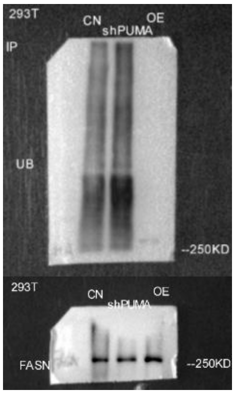

Fig.6(B)

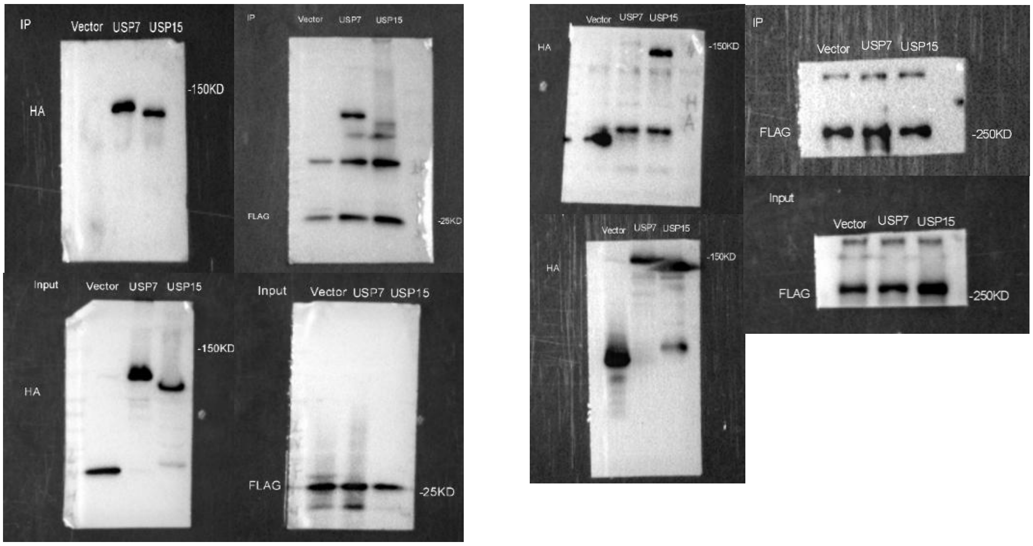

Fig.6(C)

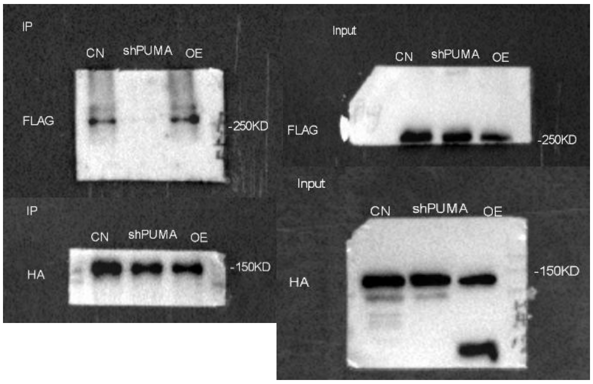

Fig.6(D)

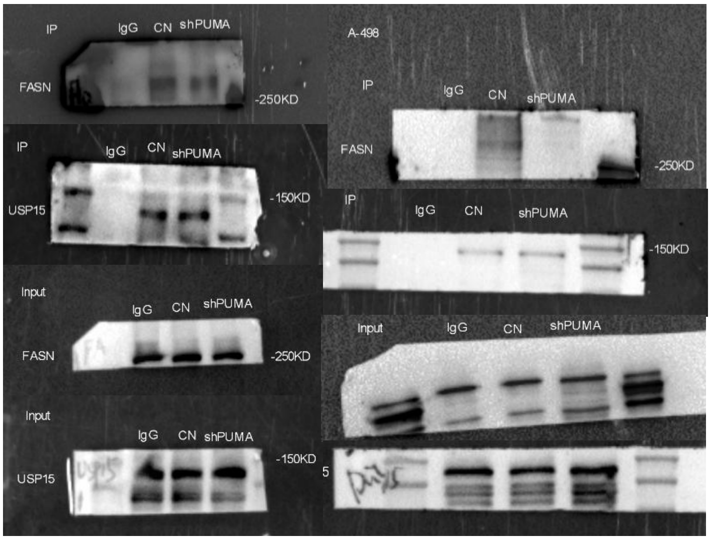

Fig.6(E)

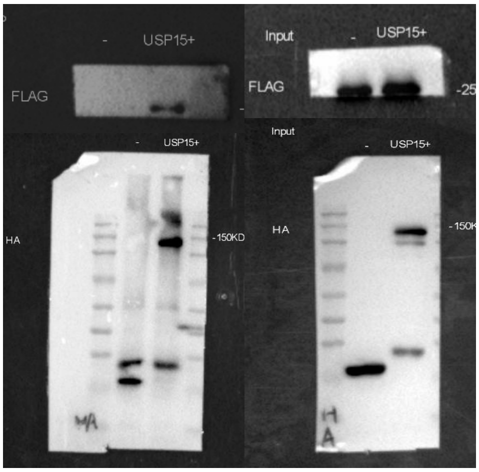

Fig.6(F)

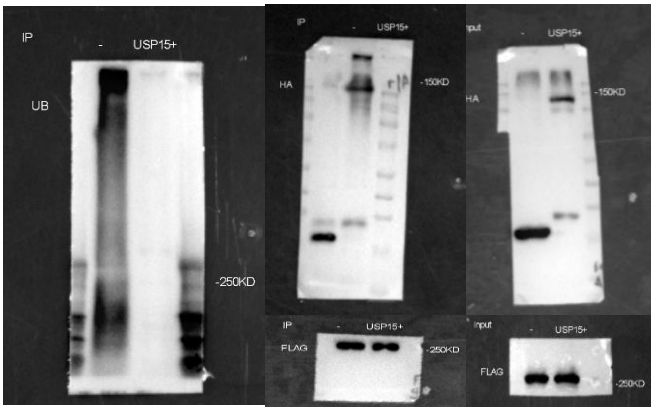

Fig.6(G)

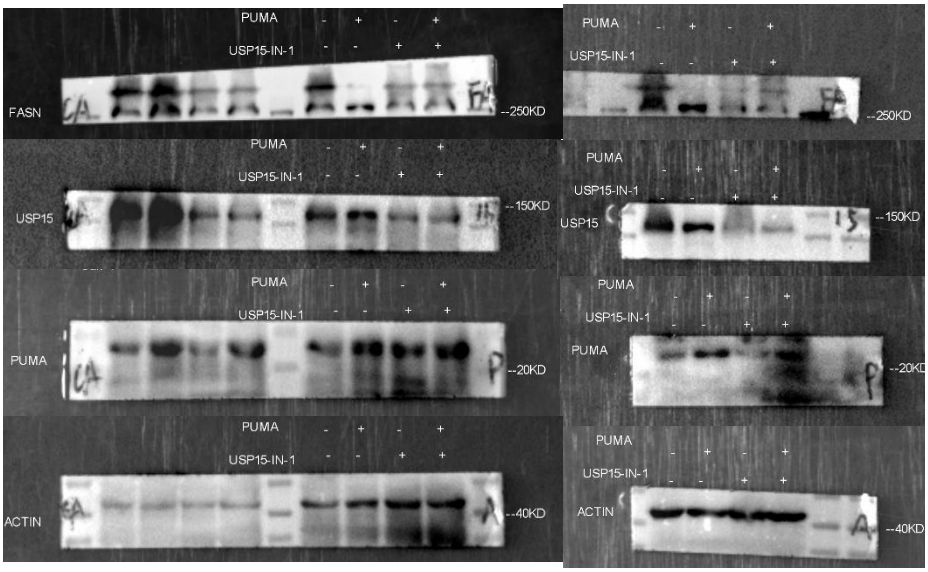

Fig.S1(B)

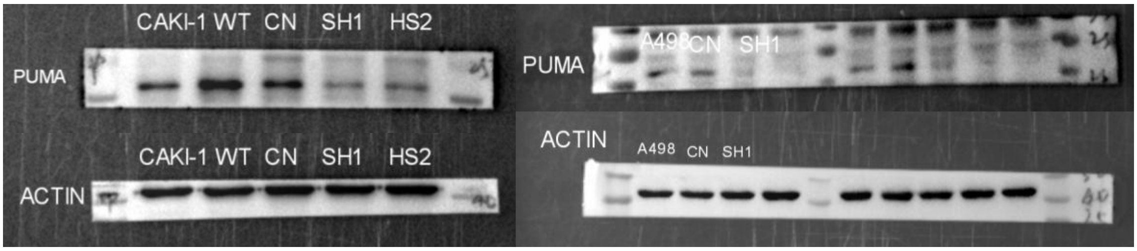

Fig.S1(D)

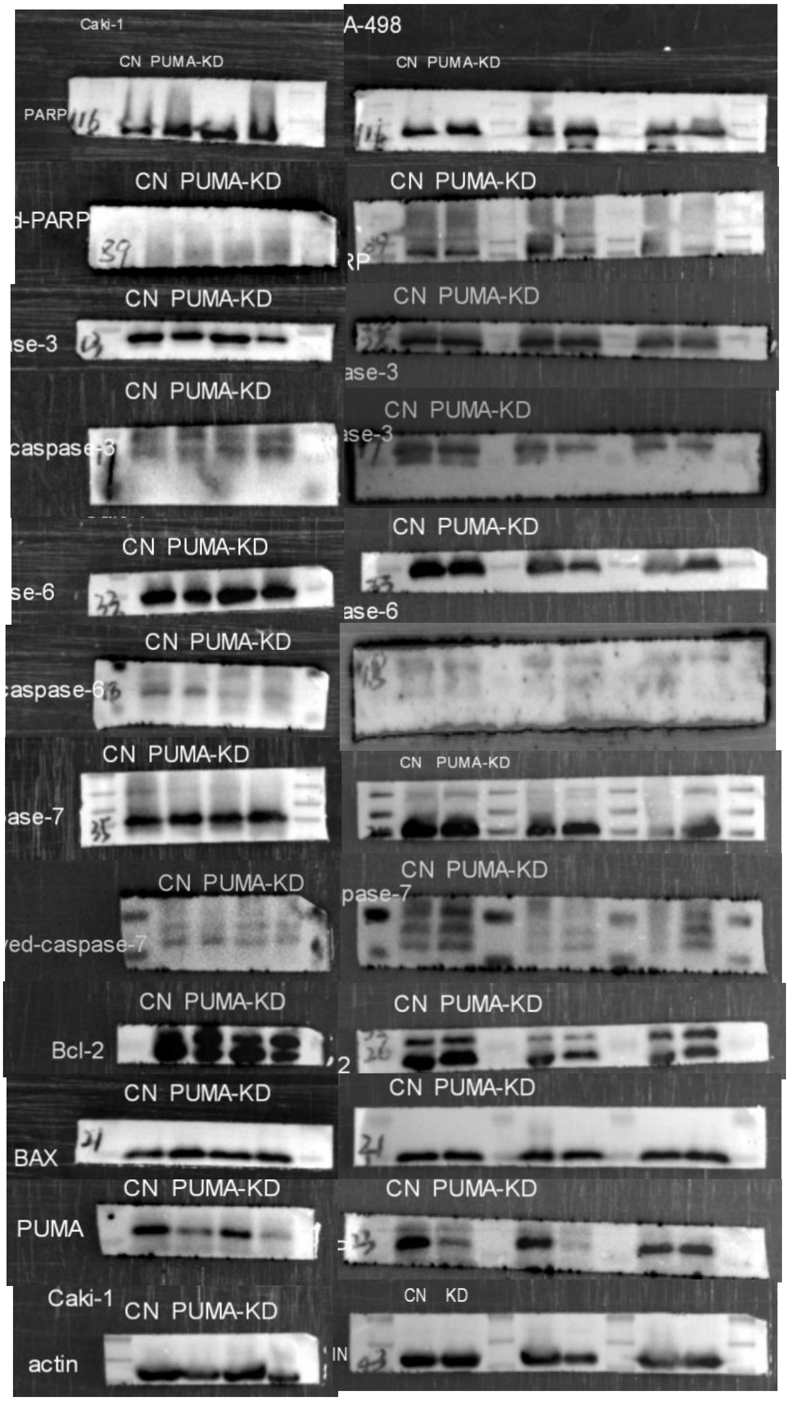

Fig.S1(E)

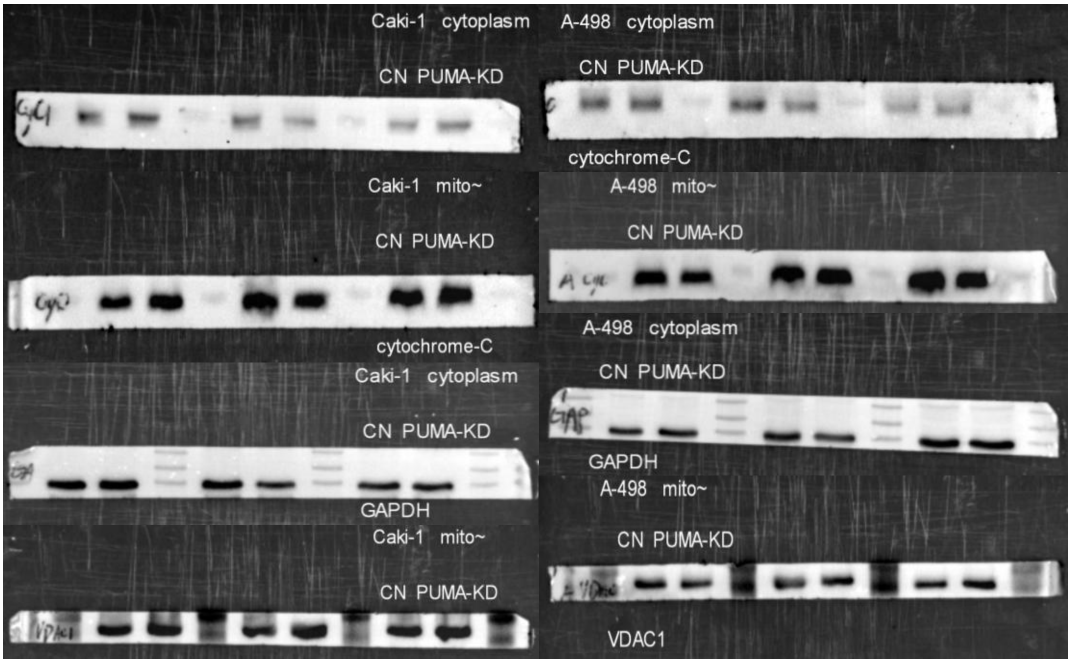

Fig.S2(F)

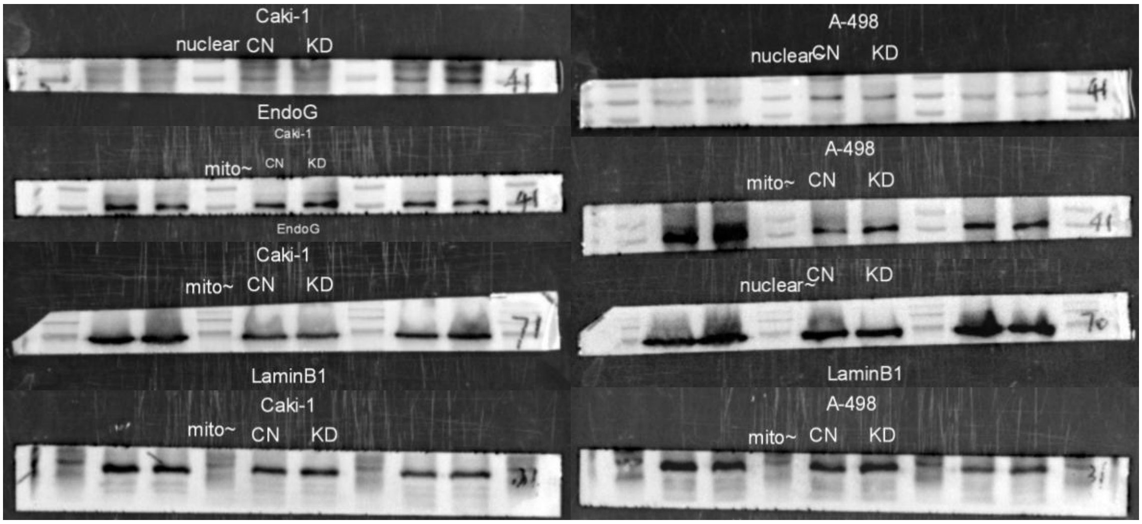

Fig.S2(G)

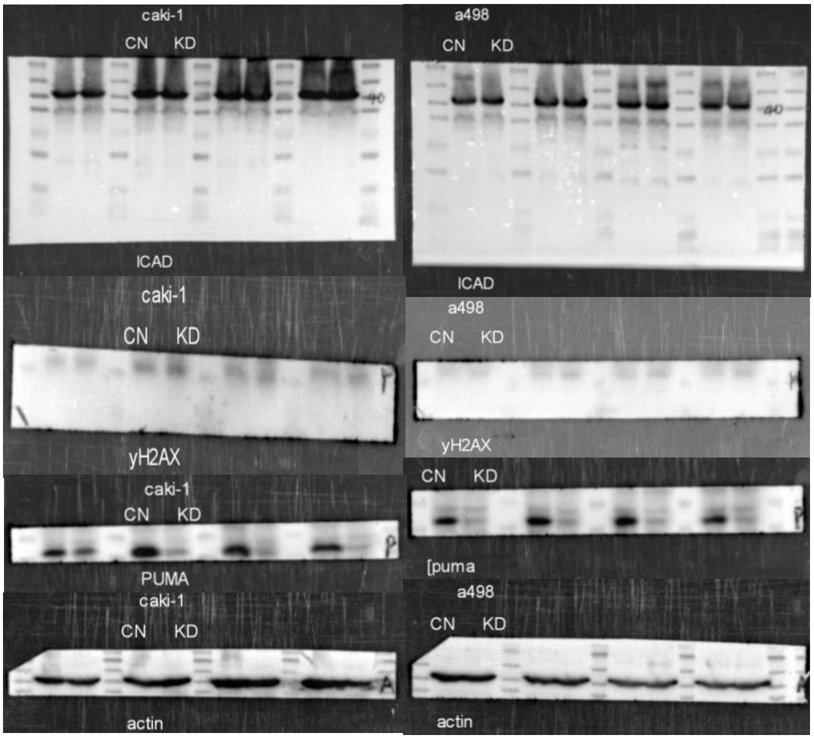

Fig.S2(H)

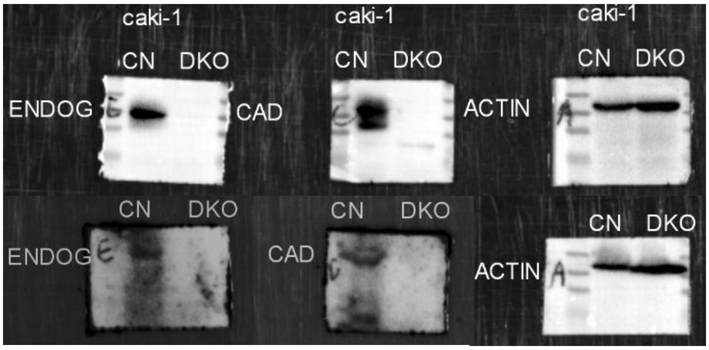

Fig.S2(J)

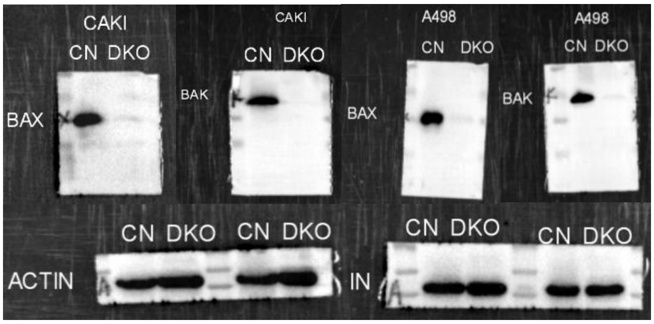

Fig.S3(A)

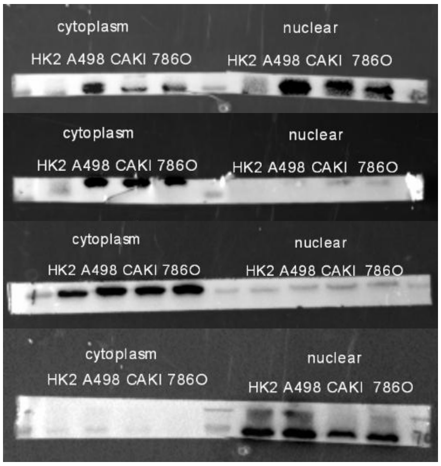

Fig.S3(B)

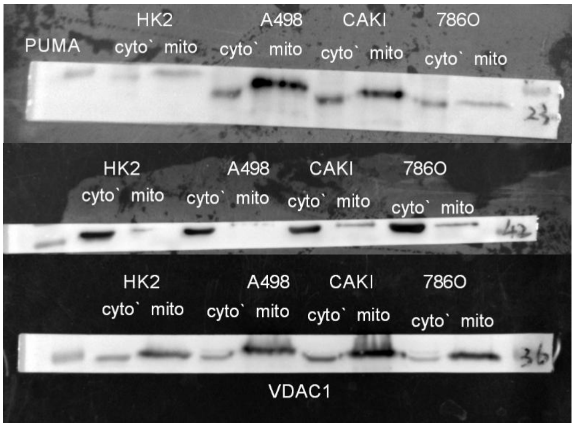

Fig.S4(H)

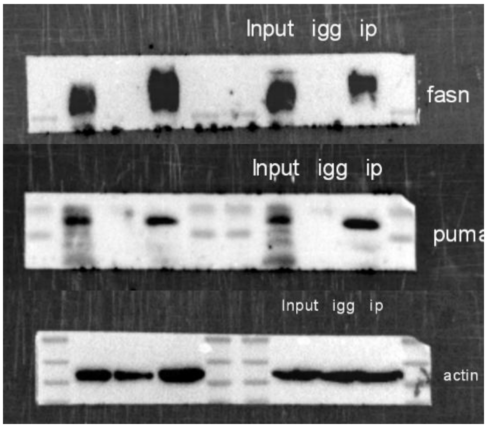

Fig.S6(C)

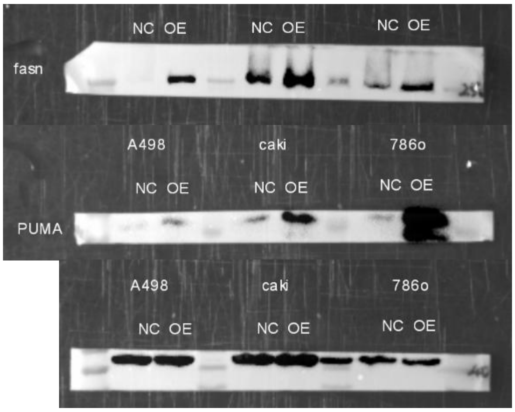

Fig.S6(E)

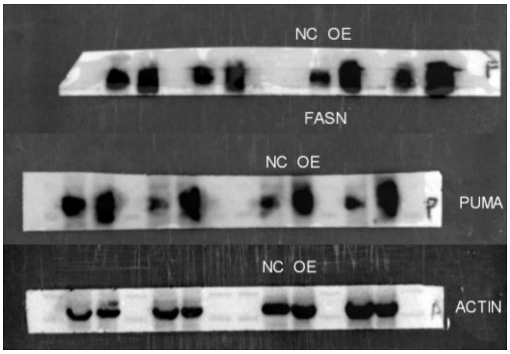

Fig.S6(F)

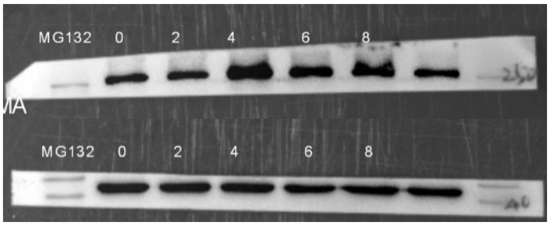

Fig.S6(G)

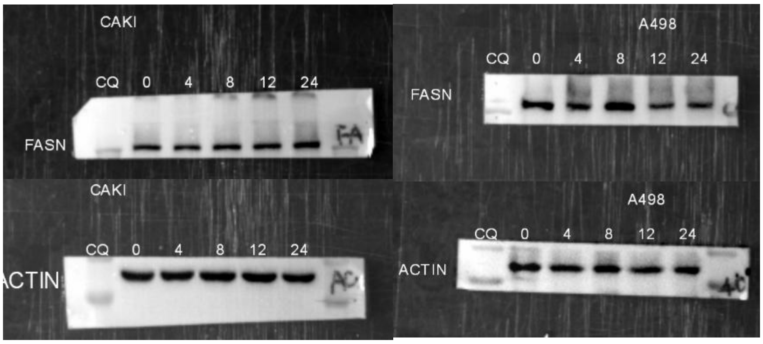

Fig.S6(H)

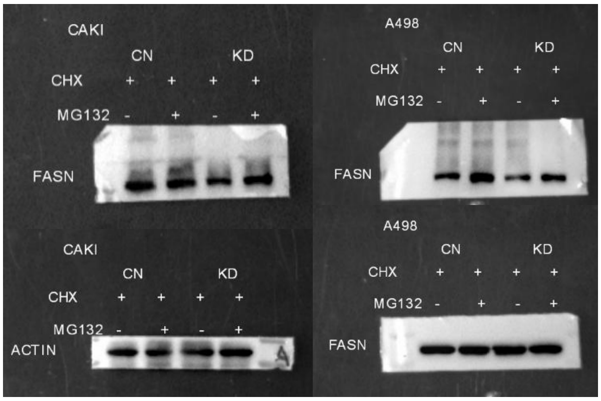

Fig.S6(I)

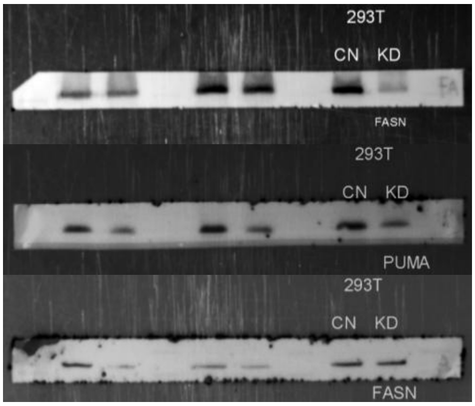

Supplement: Supplementary file 3 — Original western blot [file 41419_2025_7782_MOESM3_ESM.pdf]
